# Gut microbial Nordihydroguaiaretic acid suppresses macrophage pyroptosis to regulate epithelial homeostasis and inflammation

**DOI:** 10.1080/19490976.2025.2518338

**Published:** 2025-07-01

**Authors:** Jun Wang, Huishi Tan, Ziwen Ye, Senhui Weng, Yanqiang Shi, Jiahui Xu, Hongbin Liu, Jierui Li, Linwen Huang, Luyue Zhai, Huishan Luo, Zelong Lin, Cailing Zhong, Jing Tang, Zezheng Wang, Haiyan Zhang, Beiping Zhang, Chongyang Huang

**Affiliations:** a Department of Gastroenterology, The Second Affiliated Hospital of Guangzhou University of Chinese Medicine (Guangdong Provincial Hospital of Chinese Medicine), Guangzhou, China; b Guangdong Provincial Key Laboratory of Chinese Medicine for Prevention and Treatment for Refractory Chronic Diseases; c State Key Laboratory of Dampness Syndrome of Chinese Medicine; d Guangdong Provincial Key Laboratory of Clinical Research on Traditional Chinese Medicine Syndrome; e State Key Laboratory of Traditional Chinese Medicine Syndrome/Department of Gynecologic Oncology, Guangzhou, China; f Department of Gastroenterology and Hepatology, Guangzhou First People’s Hospital, School of Medicine, South China University of Technology, Guangzhou, China; g School of Nursing, Guangdong Pharmaceutical University, Guangzhou, China; h Institute of Dermatology and Venereology, Dermatology Hospital, Southern Medical University, Guangzhou, China; i Department of Gastroenterology, The Second Affiliated Hospital of Guangzhou Medical University, Guangzhou, China; j Guangdong Provincial Key Laboratory of Gastroenterology, Institute of Gastroenterology of Guangdong Province, Department of Gastroenterology, Nanfang Hospital, Southern Medical University, Guangzhou, China; k The First Affiliated Hospital, Faculty of Medical Science, Jinan University, Guangzhou, China

**Keywords:** Aging, gut microbiota, IBD, NDGA, macrophage pyroptosis

## Abstract

**Background:**

Aging is associated with increased severity of inflammatory bowel disease (IBD). Gut senescence and altered environmental factors contribute to changes in the intestinal metabolome, particularly in frail older individuals. However, the role of age-associated dysbiosis, characterized by a decline in beneficial gut microbiota and their metabolites, in exacerbating IBD remains unclear.

**Methods:**

To investigate the impact of aging-associated dysbiosis on colitis development, we employed fecal microbiota transplantation (FMT) in wild-type and IL-10-deficient mice. Aged mice were treated with gut microbiota from either young or aged mice and then subjected to dextran sulfate sodium (DSS) to induce experimental colitis. 16S rDNA sequencing and metabolomics were used to analyze microbial and metabolite profiles. Single-cell RNA sequencing (scRNA-seq) was performed to characterize lamina propria CD45^+^ immune cell composition.

**Results:**

Aged mice receiving microbiota from young mice exhibited less severe colitis than those receiving microbiota from aged mice, as evidenced by reduced disease activity, weight loss, and colonic shortening. Besides, aged mice displayed a significant decrease in the *Lactobacillus* population, accompanied by a reduction in Nordihydroguaiaretic acid (NDGA) levels. Decreased fecal NDGA levels were also observed in both IBD patients and elderly individuals. Administration of NDGA alleviated experimental colitis by downregulating the GSDMD/NR4A1/NLRP3 axis-mediated macrophage pyroptosis. Deletion of GSDMD in macrophages significantly diminished the protective effect of NDGA on colitis.

**Conclusions:**

Our findings demonstrate that aging is associated with dysbiosis and reduced NDGA production, which increases susceptibility to intestinal inflammation. Gut microbial NDGA exhibits potential anti-inflammatory activity in colitis, suggesting a promising therapeutic target for aged-related IBD.

## Background

Inflammatory bowel disease (IBD), encompassing Crohn’s disease (CD) and ulcerative colitis (UC), is a chronic intestinal disorder characterized by a dysregulated immune system.^1,2^ A national study revealed that over 10% of new IBD cases occur in patients aged 60 or older, who are classified as elderly IBD patients.^3,4^ The proportion of elderly IBD patients is increasing and is projected to continue rising in the coming years.^5,6^ While IBD in the elderly shares similarities with younger individuals, it is associated with a higher incidence of heart disease, infections, cancers, and IBD-related mortality.^7^ This is likely attributable to the unique challenges posed by gut senescence, including compromised intestinal barrier function, impaired resolution of intestinal inflammation, and reduced diversity of the resident microbiota.^8^ Emerging evidence suggests that an impaired intestinal barrier in the elderly significantly influences susceptibility to IBD, primarily by modulating the intestinal immune response and the gut microbiome.^9,10^

At homeostasis, the intestine houses a well-balanced gut microbiome that is essential for host metabolism and immunity.^11,12^ Aging induces dysbiotic gut microbiota, characterized by a decrease in *Firmicutes* (including *Lactobacillus*) and an increase in *Proteobacteria*, similar to the profile observed in IBD patients.^13–15^ Therefore, it is widely speculated that the high mortality rate in elderly IBD patients is linked to dysbiosis.

Gut microbiota-derived metabolites contribute to maintaining intestinal epithelial homeostasis by interacting with intestinal resident immune cells, including intestinal macrophages.^16–18^ Emerging evidence highlights microbial capacity to metabolize dietary phenolics, such as lignans and polyphenols, into bioactive compounds that critically modulate intestinal immune responses. For instance, microbial enzymatic processes, including deglycosylation and reduction, transform inert dietary phenolics into metabolites like urolithins, equol, and phloroglucinol, which exhibit immunomodulatory properties by regulating macrophage polarization, cytokine secretion, and epithelial barrier integrity.^19,20^ Macrophages play a crucial role in defending against pathogens, wound healing, and regulating inflammation.^21,22^ Gasdermin D (GSDMD)-induced pyroptosis serves as a host defense mechanism against microbial invasion and triggers robust inflammatory responses.^23,24^ By forming transmembrane pores, GSDMD facilitates the release of cytokines, such as IL-1β, and damage-associated molecular patterns (DAMPs), thereby initiating a robust immune response.^25,26^

Age-related shifts in microbial composition, such as the depletion of *Lactobacillus* and *Bifidobacterium* species, are associated with reduced enzymatic activity necessary for phenolic biotransformation.^15,27^ This functional decline likely disrupts the production of immunoregulatory metabolites, exacerbating inflammaging and compromising mucosal immunity.^28^ Consequently, the loss of microbial metabolic capacity in the elderly may represent a critical mechanism underlying the heightened susceptibility to immune-mediated disorders, including IBD.

In the present study, fecal microbiota transplantation (FMT) was conducted to investigate the role of aged-associated dysbiosis in colitis development. We discovered that aging significantly influences the composition of the intestinal microbiota and metabolome, characterized by a substantial decline in *Lactobacillus* and fecal Nordihydroguaiaretic acid (NDGA). Conversely, exogenous NDGA supplementation ameliorated experimental colitis by attenuating intestinal inflammation. In macrophages, NDGA inhibited GSDMD/NR4A1/NLRP3-mediated pyroptotic cell death and the inflammatory response. Our study reveals the therapeutic potential of NDGA in IBD and elucidates the underlying molecular mechanisms, providing novel intervention strategies for IBD treatment.

## Materials and methods

### Antibodies and reagents

NDGA (S3984) was purchased from Selleck. Lipopolysaccharides (297–473–0), vancomycin (1404–93–9), neomycin sulfate (1405–10–3), metronidazole (443–48–1), and ampicillin (69–52–3) were obtained from Sigma-Aldrich. Recombinant mouse GM-CSF (96–315–03–20) was procured from Peprotech. ATP solution (C0550) was purchased from Solarbio. All reagents were used according to the manufacturer’s instructions.

For immunoblotting and immunostaining experiments, the following antibodies were used: anti-ZO-1 (GB111981), anti-Occludin (GB111401), anti-F4/80 (GB11027), anti-IL-1β (GB11113), anti-MUC2 (GB11344), and PAS staining solution (G1008) were purchased from Servicebio; anti-GSDMD (ab209845) and anti-NR4A1 (Nur77, ab153914) were purchased from Abcam; anti-Caspase1/p20/p10 (22915–1-AP) was purchased from Proteintech; and anti-NLRP3 (#15101) was purchased from Cell Signaling Technology.

### Mouse models

Wild-type C57BL/6J mice (2 or 18 months old), IL-10-deficient mice, and GSDMD^fl/fl^Lyz2-Cre mice were obtained from Cyagen Biosciences. All mice were housed under specific pathogen-free conditions in accredited animal facilities at The Second Affiliated Hospital of Guangzhou University of Chinese Medicine. Mice were fed a standard laboratory chow diet (Research Diets D12450J; 10% fat, 70% carbohydrate, 20% protein). All animal research protocols were approved by the Institutional Animal Care and Use Committee of The Second Affiliated Hospital of Guangzhou University of Chinese Medicine (Animal Ethical Statement No: 2022042). All experiments were conducted in accordance with Chinese law regarding animal protection and the “Guide for the Care and Use of Laboratory Animals” (National Institutes of Health publication, 8th Edition, 2011).

To evaluate the therapeutic effect of NDGA on DSS-induced colitis, mice were randomly divided into 6 groups: control group, NDGA group (5 mg/kg body weight), DSS-induced colitis group, and DSS plus NDGA group (0.5, 1, 5 mg/kg body weight). The dosage of NDGA was determined with reference to the previous study.^29^ Mice in the DSS-induced colitis group and NDGA-treated DSS-induced colitis group were administered 2.5% DSS in drinking water for 7 days to induce colitis, while mice in the control group received regular drinking water. Mice in the NDGA-treated DSS-induced colitis group were administered with NDGA orally once daily for 10 consecutive days starting from 3 days before DSS administration. Body weight and disease activity index (DAI) were monitored daily. The DAI of colitis was assessed based on weight loss, stool consistency, and rectal bleeding.

Homozygous IL-10-KO mice were randomly divided into control (PBS) and NDGA (5 mg/kg body weight, every 2 days) treatment groups. The development of spontaneous colitis was monitored through weekly assessments of body weight, stool consistency, and histological analysis of colon tissues.

### Human subjects and ethics declaration

Fecal samples were collected from 50 IBD patients and 38 healthy donors. Informed consent was obtained from all participants. All diagnoses and clinical disease activity assessments were based on a standard combination of clinical, endoscopic, histological, and radiological criteria. Endoscopic colonic mucosal biopsy samples were collected from active and remission UC patients attending the Department of Gastroenterology. All intestinal biopsies were collected from consenting individuals during routine endoscopy. All individuals provided informed written consent to participate. All experiments were performed in accordance with the ethical committee of The Second Affiliated Hospital of Guangzhou University of Chinese Medicine and the clinical experimental guidelines of Guangzhou University of Chinese Medicine (approval number: BF2023–008–01). Related experiments were conducted in accordance with the Declaration of Helsinki. Demographic characteristics are shown in Table 1.

**Table 1.** Clinical patient characteristics.

	Healthy Volunteers (Young)	Healthy Volunteers (aged)	UC	CD
Number	20	18	25	25
Gender (m/f)	10/10	9/9	12/13	18/7
Age (year)	25 (18–29)	60 (55–70)	55 (31–68)	30 (18–47)
Mayo score	–	–	3(1–6)	–
CDAI score	–	–	–	100(22–487)

Values are reported as mean and (range). CDAI: Crohn’s Disease Activity Index.

### Fecal microbiota transplantation

FMT was performed as previously described.^30^ FMT was conducted to assess the impact of age-associated dysbiosis on colitis development. Fresh fecal samples were collected from donor mice of different ages, and fecal suspensions were prepared under sterile conditions. Recipient mice were pretreated with antibiotics to deplete their endogenous microbiota before FMT. Four antibiotics (ampicillin 4 mg/20 g, neomycin sulfate 4 mg/20 g, metronidazole 4 mg/20 g, and vancomycin 2 mg/20 g) were administered intragastrically once daily for 5 days to deplete the gut microbiota. Feces from donor mice were collected and resuspended in PBS at 0.125 g/mL, and then 0.15 mL of this suspension was administered to mice by oral gavage once daily for 5 days.

*Lactobacillus johnsonii*, *Ligilactobacillus murinus*, *and Lactobacillus reuteri* freeze-dried powder were purchased from BeNa Culture Collection (Beijing, China). A single colony was picked into Man Rogosa Sharpe (MRS) Broth (Solarbio, Beijing, China), and the bacterial liquid was incubated at 37°C under anaerobic conditions. After 16–24 hours of growth, the OD_600_ of the cultures reached 0.5–0.6, indicating the logarithmic phase with a colony count of 5 × 10^8^ CFU/mL. Aliquots containing 5 × 10^8^ CFU/mouse/day were resuspended in 200 µL PBS and administered to mice by gavage for 7 days. Control mice received an equal volume of sterile PBS by gavage.

### Histological analysis

The colons were excised from euthanized mice, fixed in 4% paraformaldehyde, dehydrated, and embedded in paraffin. Paraffin blocks were sectioned into 4-μm thick tissue sections for further analysis. Tissue sections were stained with hematoxylin and eosin (H&E) to reveal cellular architecture and tissue composition. For immunofluorescence (IF) staining, sections were deparaffinized, rehydrated, and subjected to heat-induced epitope retrieval using citrate buffer. Sections were blocked with 5% BSA for 30 minutes and incubated with primary antibody as per the manufacturer’s instructions. Subsequently, sections were incubated with HRP-conjugated secondary antibody (ZSGQ-Bio, Beijing, China) at room temperature for 1 hour and stained with diaminobenzidine (Boster Bio, Wuhan, China) for 3 minutes. Hematoxylin counterstaining was performed, and the results were observed under a microscope (Olympus, Tokyo, Japan).

### Isolation and culture of Bone marrow-derived macrophages (BMDMs)

Isolation and culture of BMDMs were performed as previously described.^31^ Mouse primary BMDMs were isolated from bone marrow progenitors flushed from femurs and tibias. During differentiation, cells were cultured in DMEM containing 15% FBS with supplements, including 30 ng/mL recombinant mouse granulocyte-macrophage colony-stimulating factor (GM-CSF) (PeproTech, Rocky Hill, USA). After 4 days of incubation, the supernatant was removed and replaced with a fresh, complete medium containing GM-CSF. After 7 days of incubation, the BMDMs were used for further experiments. For activation of the macrophage pyroptosis, BMDMs and Raw264.7 cells were concurrently stimulated with LPS (100 ng/mL) for 4 hours, ATP (5 mM) for 30 minutes, and different concentrations of NDGA.

### Immunoprecipitation (IP)

BMDMs were cultured with LPS (100 ng/mL) for 4 hours, followed by ATP (5 mM) stimulation for 30 minutes. For the LPS+ATP plus NDGA group, BMDMs were treated with LPS and NDGA for 4 hours prior to ATP exposure. For immunoprecipitation, BMDMs lysates were prepared and incubated with 1 µg of control IgG or anti-NLRP3 antibody under constant rotation at 4°C for 1 hour, followed by overnight incubation with 30 µL protein A/G plus agarose (sc-2003, Santa Cruz, USA). The agarose beads were washed 5 times with IP wash buffer. The immunocomplexes were immunoblotted with an anti-NR4A1 antibody.

### Microbiota 16S rDNA gene sequencing

Microbial composition analysis was performed using 16S rDNA gene sequencing. DNA was extracted from stool samples. The 16S rDNA gene, a conserved region within the bacterial genome (specifically the V4 region), was amplified using barcode-tagged primers. The forward primer sequence was 5’-GTGTGYCAGCMGCCGCGGTAA-3' (V4F), and the reverse primer sequence was 5’-CCGGACTACNVGGGTWTCTAAT-3' (V4R). All samples were subjected to paired-end sequencing on the Illumina HiSeq PE250 platform. Data analysis and graphical representation were conducted utilizing a specialized online platform dedicated to next-generation sequencing data analysis and visualization, available at https://www.omicsmart.com/, provided by GENE DENOVO from Guangzhou, China.

### Untargeted metabolomics analysis

For sample preparation, freeze-dried leaves were pulverized using a mixer mill (MM 400, Retsch) with a zirconia bead for 1.5 minutes at 30 hz. A 100 mg powder sample was extracted overnight at 4°C in 1.0 mL of 70% aqueous methanol. After centrifugation at 10,000 g for 10 minutes, the extracts were cleaned using a CNWBOND Carbon-GCB SPE Cartridge (250 mg, 3 mL) and filtered through a 0.22 μm membrane before LC-MS analysis.

For HPLC analysis, a Shim-pack UFLC SHIMADZU CBM30A system coupled with an Applied Biosystems 6500 Q TRAP mass spectrometer was employed. A Waters ACQUITY UPLC HSS T3 C18 column was used, and the mobile phase consisted of water and acetonitrile, both containing 0.04% acetic acid. The flow rate was set at 0.40 mL/min at a temperature of 40°C, with an injection volume of 2 μL. The effluent was directed to an ESI-triple quadrupole-linear ion trap (Q TRAP)-MS system.

The mass spectrometry analysis was performed using an API 6500 Q TRAP in positive ion mode, controlled by Analyst 1.6.3 software. Key parameters included a source temperature of 500°C, an ion spray voltage of 5500 V, and specific pressures for the ion source gases. The instrument was tuned and mass calibrated, with QQQ scans acquired through MRM experiments. Detection was focused on specific metabolites during their elution periods, optimizing collision energy and declustering potential for individual transitions.

### Single-cell isolation, library preparation, and sequencing

CD45-positive leukocytes were isolated from the lamina propria of the dextran sulfate sodium (DSS)-treated colon on day 7. Cell viability was assessed by trypan blue exclusion, with all treatment groups exhibiting >90% viability. Cells were passed through a 30 μm nylon mesh to obtain a single-cell suspension. CD45-positive cells were enriched using MACS MicroBeads and MACS separators. Cell counts were performed using a Countess II Automated Cell Counter. Cell concentrations were adjusted to at least 1000 cells/μL. Single-cell isolation and RNA library preparation were performed using the 10× Chromium Single-Cell 3’ RNA-seq Kit (version 3 Chemistry, 10× Genomics), following the manufacturer’s instructions. Libraries were sequenced on an Illumina sequencing platform at OMICSHARE, yielding >100,000 reads per cell. Data analysis and graphical representation were conducted utilizing a specialized online platform dedicated to next-generation sequencing data analysis and visualization, available at https://www.omicsmart.com/, provided by GENE DENOVO from Guangzhou, China.

### NDGA detection and analysis

LC-MS/MS mass spectrometry was used for the absolute quantification of NDGA. Solid-phase extraction was performed as follows: the SPE solid-phase extraction column was activated with 3 mL of water followed by 3 mL of methanol. The supernatant was loaded onto the column at a flow rate of ≤1 mL/min. The column was rinsed with 3 mL of water and 3 mL of 10% methanol (Sigma-Aldrich) in water. Finally, the analytes were eluted with 1 mL of methanol. The eluent was concentrated to dryness, re-dissolved in 0.60 mL of 80% methanol-water, vortexed for 1 minute, centrifuged at 12,000 rpm for 10 minutes, and the supernatant was subjected to LC-MS/MS analysis. All Total Ion Chromatograms (TICs) have been uploaded as a ZIP file.

### Quantitative real-time PCR

Total RNA was extracted using TRIzol reagent (ACCURATE, AG21102) and reverse transcribed into cDNA using a reverse transcription kit (TOYOBO, FSQ-101) according to the manufacturer’s instructions. Quantitative real-time PCR (qRT-PCR) was performed on a Roche LightCycler 96 system using primer sequences obtained from PrimerBank and previous studies, as listed in Table 2.

**Table 2.** Primers for RT-PCR.

Gene	Left primer(5’−3’)	Right primer(5’−3’)
*m*-18s	CGATCCGAGGGCCTCACTA	AGTCCCTGCCCTTTGTACACA
m-IL-1β	GGTCAAAGGTTTGGAAGCAG	TGTGAAATGCCACCTTTTGA
m-TNF-α	ACCACGCTCTTCTGTCTACTGA	AGCTGCTCCTCCACTTGGT
m- IL-6	TGATGCACTTGCAGAAAACA	ACCAGAGGAAATTTTCAATAGGC
m-Ccl3	ACCATGACACTCTGCAACCA	GTGGAATCTTCCGGCTGTAG
m-Ccl4	CATGAAGCTCTGCGTGTCTG	GAAACAGCAGGAAGTGGGAG
m-Cxcl1	ACCCAAACCGAAGTCATAGC	TCTCCGTTACTTGGGGACAC
m-Cxcl2	CGGTCAAAAAGTTTGCCTTG	TCCAGGTCAGTTAGCCTTGC
m-Nos2	TAGGCAGAGATTGGAGGCCTTG	GGGTTGTTGCTGAACTTCCAGTC

m, mouse; IL, interleukin; TNF, tumor necrosis factor; Ccl, chemokine (C-C motif) ligands; Cxcl, chemokine (C-X-C motif) ligands.

### Western blot

Intestinal tissue and cell proteins were extracted using RIPA lysis buffer (Thermo Scientific) according to the manufacturer’s instructions. Western blot analysis was performed using a color PAGE gel rapid preparation kit from Epizyme Biotech (Shanghai, China). Proteins were transferred onto PVDF membranes using the WIX-fast BLOT semi-dry blotting system from WIX (Beijing, China). Primary antibodies were applied to the membranes at the recommended dilutions. Protein bands were detected using ECL chemiluminescent substrate from Beyotime Biotech (Shanghai, China), and the resulting signals were captured and analyzed using a Bio-Rad gel imaging system.

### Statistical analysis

Unless otherwise indicated, statistical analyses were performed using GraphPad Prism software 8.0. One-way ANOVA and post-hoc tests were employed to analyze the data from multiple groups. A two-tailed Student’s *t*-test was used to analyze the data between two groups. Values of *p* < 0.05 were considered statistically significant.

## Results

### Aged-associated dysbiotic microbiota aggravates experimental colitis

It is well-established that aging worsens colitis and IBD disease activity.^32^ We hypothesized that aged-related alterations in the gut microbiota contribute to this unique phenotype. Consequently, FMT was performed to elucidate the role of age-related dysbiosis in the development of colitis (Figure S1A). Mice that received gut microbiota transplantation from aged mice (FMT-18 M) exhibited more severe DSS-induced colitis than mice that received young gut microbiota (FMT-2 M), as evidenced by lower survival rates, increased body weight loss, and colon length shortening (Figure S1B-G). Histological examination of colon sections from the FMT-18 M group revealed widespread intestinal epithelial destruction and extensive transmural expansions of inflammatory cell infiltrations following DSS administration, compared to the FMT-2 M group (Figure S1H-K). IL-10-deficient mice develop spontaneous colitis due to host immune dysregulation, which closely mimics the pathogenesis of human IBD.^33^ Here, a similar effect was observed in IL-10-deficient mice given elderly microbiota transplantation, as evidenced by increased weight loss, colonic shortening, and histopathological scores (Figure S2A-K). These findings suggest that aged-associated gut dysbiosis significantly exacerbates intestinal inflammation in IBD models.

**Figure 1.**
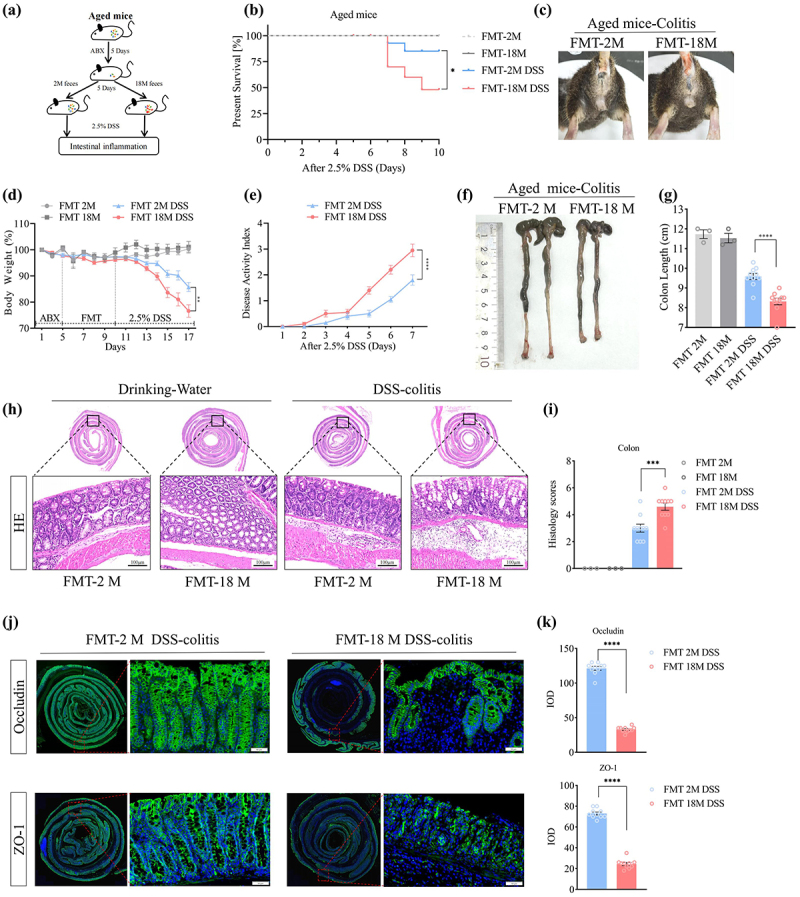
Gut microbiota remodeling alleviates experimental colitis in aged mice. (a) Experimental design of fecal bacterial transplantation; (b) The overall survival curves of DSS-treated mice; (c) bloody stools of DSS-treated mice; (d) body weight changes were daily monitored after DSS administration; (e) DAI were scored in DSS-treated mice; (f–g) mice were euthanized on day 7, and colon lengths were measured; (h-i) representative images of the histological-examined colon sections; (j-k) representative immunofluorescence images of tight junction proteins (occludin, ZO-1) in the DSS-treated colonic tissues and its quantitative analysis.

We next sought to investigate whether intestinal microbiota remodeling in aged mice could improve colitis remission. Aged mice received fecal microbiota from young and aged mice before being subjected to experimental colitis modeling (Figure 1(a)). Here, gut microbiota remodeling in aged mice improved body weight loss, survival rate, and decreased disease activity index in response to DSS treatment (Figure 1(b–g)). Furthermore, aged mice that received fecal microbiota from young mice exhibited significantly less severe intestinal histological damage and barrier dysfunction than those that received microbiota from aged mice (Figure 1(h–k)). These results suggest that specific core microbiota communities with anti-inflammatory properties are lost with aging.

(Data in each group were expressed as mean ± SEM, *n* = 8. *p < 0.05, **p < 0.01, ***p < 0.001, ****p < 0.0001; analyzed by one-way ANOVA with Holm-Sidak post hoc tests. The survival rates of DSS-treated mice were analyzed using the Kaplan-Meier method with the logrank test. Data shown are representative of three independent experiments.)

### Aging alters the gut microbiota community and leads to reduced fecal NDGA

**Figure 2.**
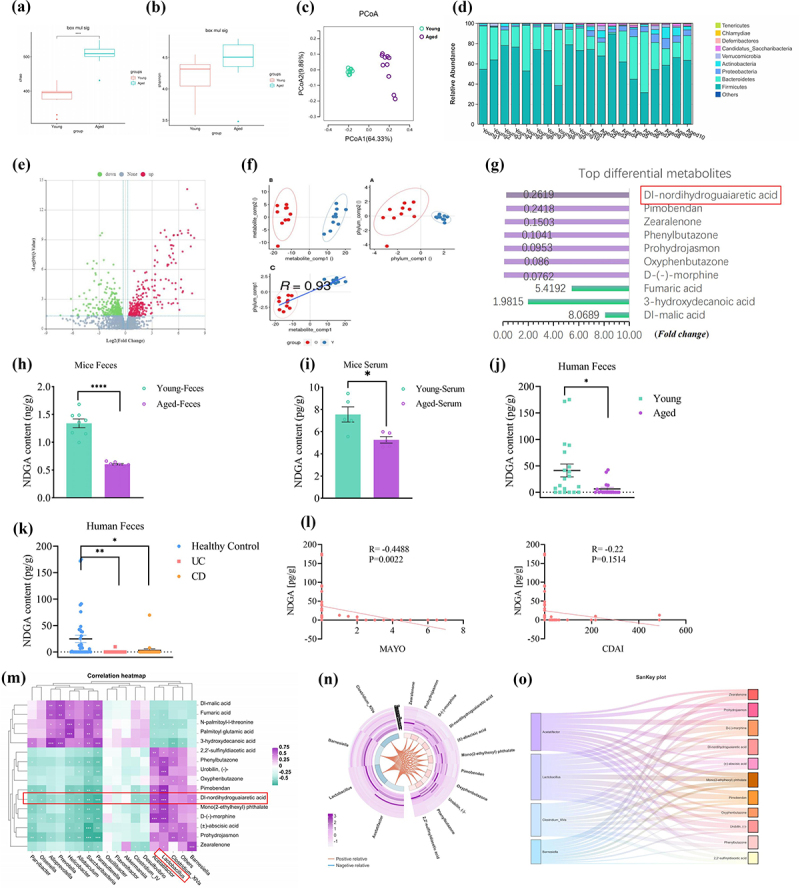
Aging alters the gut microbiota community and leads to reduced fecal NDGA. (a–b) Box plots of alpha-diversity indices (Shannon and Chao); (c) principle coordination analysis (PCoA) between group Olderly(O) and group young(Y) microbiota colonies based on OTU abundance;(d) relative gut microbiota composition at the phylum level;(e) volcano plot showing the differential metabolites of group O and Y; (f) Principal component analysis (PCA) showing the correlation between the differential metabolites and the gut microbiota composition at the phylum level; (g) differential metabolites of group O and Y (TOP 10 differential metabolites); (h-i) fecal and serum NDGA levels were detected by targeted metabolomics in mice; (j-k) fecal NDGA levels were detected by targeted metabolomics in human; (l) correlation analysis between fecal NDGA levels and the clinical severity of IBD; (m) correlation heatmaps illustrate the relationship between differential strains and metabolites; (n-o) sector and Sankey plots demonstrate the association between differential strains and metabolites. (for 16s-seq and untargeted metabolomics, *n* = 10. For targeted metabolomics in mice, *n* = 5-8. For targeted metabolomics in human, young *n* = 20, aged *n* = 18, healthy control *n* = 38, UC *n* = 25 and CD *n* = 25).

The differences in gut microbiota and metabolites between the young and aged groups were then investigated using 16S rDNA gene sequencing and metabolomics. Alpha diversity (Chao1 and Shannon indices) was higher in aged mice compared to young mice (Figure 2(a–b)). Additionally, principal coordinate analysis (PCoA) revealed a clear separation between the microbial compositions of the two groups (Figure 2(c)). Microbial composition analysis showed that the relative abundances of *Verrucomicrobiota* and *Proteobacteria* were significantly higher in the aged group, while *Firmicutes* abundance was lower (Figure 2(d)). Untargeted metabolomics data revealed significant shifts in numerous metabolites between the two groups, correlated with aged-associated microbial community changes (Figure 2(e–f)).

NDGA, a lipoxygenase inhibitor with potential anti-aging and anti-inflammatory properties, was found to be less abundant in the feces of the aged group compared to the young group (Figure 2(g)). The reduction of fecal and serum NDGA in aged mice was further confirmed by mass spectrometry (MS) experiments (Figure 2(h-i)). Notably, a significant reduction in fecal NDGA levels was observed in aged individuals and patients with active IBD (Figure 2(j–k)). Besides, fecal NDGA was inversely correlated with Mayo scores in patients with UC (*r* = −0.4479, *p* < 0.01), but it did not correlate with Crohn’s Disease Activity Index (CDAI) in patients with CD (*r* = −0.22, *p* = 0.1514) (Figure 2(l)). A previous study reported that long-term consumption of dietary fat leads to a reduction in fecal NDGA.^34^ However, the microbial origin of fecal NDGA remains unclear. Interestingly, our data revealed a genus/species-level relationship between the gut microbiota and fecal NDGA (Figure 2(m)). Specifically, a lower amount of fecal NDGA was strongly correlated with a decreased *Lactobacillus* population (Figure 2(n–o)). Importantly, antibiotic treatment in young mice significantly reduced NDGA levels, suggesting that NDGA production largely depends on the commensal gut microbiota (Figure S3A-B). Subsequent colonization of mice with *Lactobacillus johnsonii*, *Ligilactobacillus murinus*, *and Lactobacillus reuteri* restored the total level of NDGA. These results suggest that *Lactobacillus* promotes the accumulation of NDGA in the intestine (Figure S3A-B). Next, we investigated whether *Lactobacillus* directly converts dietary polyphenols into NDGA. However, our findings indicated that NDGA was undetectable in the cultured supernatant of *Lactobacillus* populations or standard dietary components, suggesting that *Lactobacillus* does not directly mediate the generation of NDGA (Figure S3C). Instead, mice fed dietary polyphenols exhibited an increased tendency for fecal NDGA accumulation, indicating that the gut microbiota may mediate the conversion of microbial secondary metabolites to produce NDGA (Figure S3D).

### NDGA restrains intestinal inflammation in the murine model of IBD

**Figure 3.**
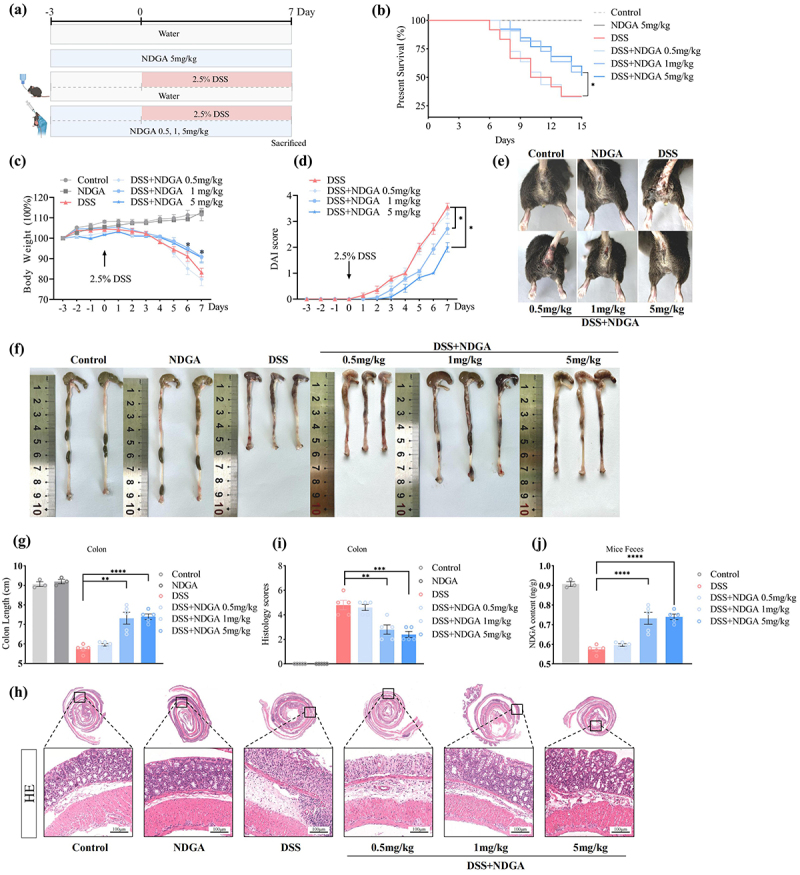
NDGA dose-dependently ameliorates DSS-induced colonic inflammation. (a) Experimental design of NDGA treatment in DSS-induced colitis; (b) the overall survival curves of DSS-treated mice; (c) body weight changes were daily monitored after DSS administration; (d) DAI was measured in DSS-treated mice; (e) bloody stools of DSS-treated mice; (f–g) mice were euthanized on day 7, and colon lengths were measured; (h–i) representative images of the histological-examined colon sections and the pathological scores were quantified; (j) fecal NDGA levels were detected by targeted metabolomics in mice.

Notably, empirical evidence from both murine and human studies has demonstrated a correlation between the depletion of specific microbial metabolites and increased disease severity.^22,35^ Considering the well-established pro-inflammatory and pro-tumorigenic roles of arachidonic acid derivatives, we postulated that a reduction in gut microbiota-derived NDGA, a compound with anti-inflammatory properties, may contribute to the progression of colitis. Consistent with this hypothesis, oral administration of NDGA exhibited a dose-dependent protective effect against DSS-induced intestinal inflammation in mice, as evidenced by a reduction in diarrhea, hematochezia, and colon shortening (Figure 3(a-g)). Notably, NDGA treatment mitigated the histopathological hallmarks of colitis, including inflammatory cell infiltration, crypt abscess formation, and epithelial damage (Figure 3(h–i)). Here, we determined that fecal levels of NDGA increased in a dose-dependent manner following NDGA administration (Figure 3(j)). Interestingly, the prophylactic administration of NDGA is less effective in reducing colitis severity compared to therapeutic dosing, suggesting that NDGA exerts its anti-inflammatory effect primarily through direct administration (Figure S4A-G).

(Data for each group are expressed as mean ± SEM, *n* = 5. *p < 0.05, **p < 0.01, ***p < 0.001, ****p < 0.0001; analyzed by one-way ANOVA with Holm-Sidak post hoc tests. The survival rates of DSS-treated mice were analyzed using the Kaplan-Meier method with the logrank test. Data shown are representative of three independent experiments).

In alignment with these findings, NDGA administration dose-dependently ameliorated the reduction in goblet cell numbers and mucus layer thickness, as assessed by Periodic Acid-Schiff (PAS) and mucin 2 (MUC2) immunostaining (Figure S5A-D). Furthermore, immunofluorescence analysis revealed a significant upregulation of zonula occludens 1 (ZO-1) and occludin (OCLN) in the NDGA-treated colon following DSS challenge, indicating that NDGA exerts a protective effect on intestinal barrier integrity (Figure S5E-H). Similarly, oral NDGA administration attenuated the progression of IL-10 knockout-induced colitis, as evidenced by improved body weight and clinical colitis scores (Figure S6A-F). Histological examination revealed a reduction in colitis severity in the NDGA-treated groups (Figure S6G-H). Collectively, these findings suggest that NDGA may offer protection against colitis induced by DSS treatment and IL-10 deficiency.

Next, we investigated whether the administration of NDGA could fully reverse the age-related microbiota-induced exacerbation of DSS-induced colitis in aged mice. Here, we found that NDGA treatment was not as effective as the administration of young microbiota, as evidenced by only partial improvements in colon length and histopathology (Fig S7A-G). These results suggest that microbial NDGA partly accounts for the beneficial effects of young microbiota in modulating gut homeostasis.

### NDGA attenuates colitis through suppression of pro-inflammatory macrophage

**Figure 4.**
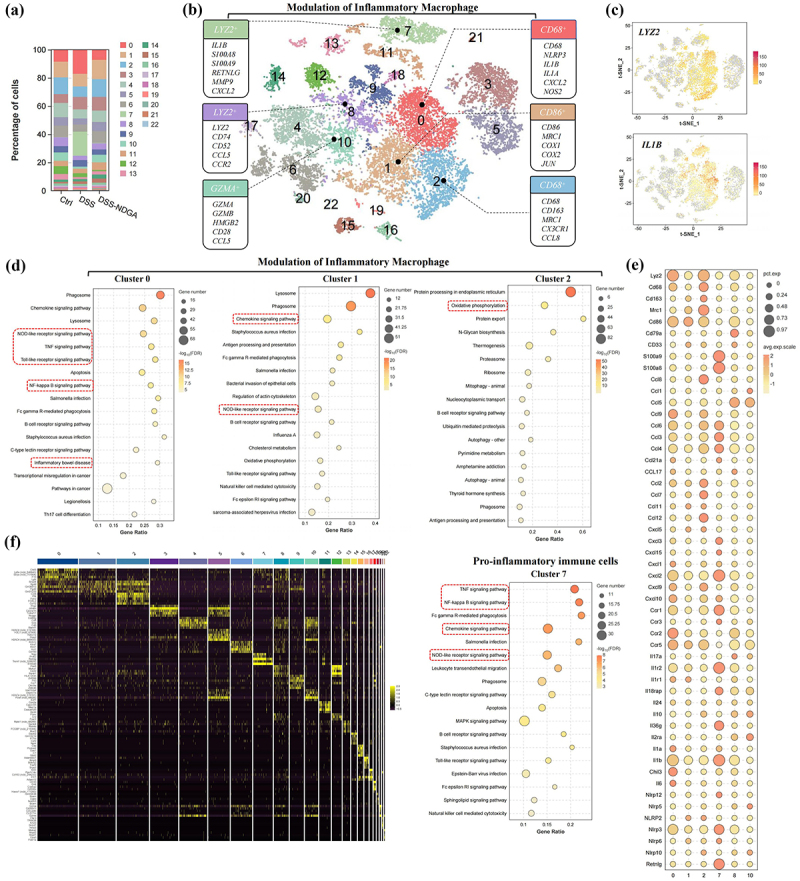
NDGA decreases the accumulation of colonic inflammatory macrophage subsets. (a) Column plots of different cell subsets in three samples; (b) tSNE mapping of cellular subpopulations (labeled datasets are inflammatory macrophage subpopulations); (c) tSNE distribution of the Lyz2 and IL-1β-positive cells; (d) KEGG pathway enrichment analysis of different macrophage subsets; (e) genes bubble maps of indicated macrophage subsets; (f) heatmap of overall differential gene expression of indicated immune cell clusters.

To gain a comprehensive understanding of the underlying mechanism by which host-microbial NDGA regulates immune responses, we performed single-cell RNA sequencing (scRNA-seq) on sorted CD45^+^ cells isolated from the intestinal lamina propria. A total of 18,687 cells were assigned to 22 distinct cell clusters using unbiased graph-based clustering (UMAP). NDGA treatment significantly altered the overall cellular composition, as evidenced by changes in the proportions of different cell clusters (Figure 4(a)). Based on the expression of marker genes (CD68 and CD86), clusters 0, 1, and 2 were identified as monocyte/macrophage populations (Figure 4(b-c)). Among these clusters, cluster 0 exhibited the highest inflammatory signature, characterized by the expression of pro-inflammatory genes such as NLRP3, IL-1β, IL-1α, and CXCL2. In contrast, cluster 2 primarily expressed genes involved in tissue repair and chemokine production, including mannose receptor C-type 1 (MRC1), CD163, and CX3CR1 (Figure S8A-D).

NDGA significantly reduced the accumulation of inflammatory macrophages in the DSS-treated colon, as well as the overall expression of pro-inflammatory cytokines (Figure S9A). Additionally, cluster 7, characterized by the expression of genes such as S100A8/9, Matrix metalloproteinase-9 (MMP-9), lipocalin 2(LCN2), C-X-C chemokine receptor 2 (CXCR2), CXCL3, leucine-rich alpha-2 glycoprotein 1 (LRG1), and Chitinase 3-like 1 (Chi3l1) was identified as a neutrophil population, which was also significantly diminished by NDGA treatment (Figure S9B-D). Pathway analysis revealed that both clusters 0 and 7 were enriched for pro-inflammatory pathways, including NOD-like receptor signaling, TNF signaling, and NF-κB signaling. In contrast, clusters 1 and 2 exhibited no significant pro-inflammatory phenotype (Figure 4(d–f)). Our findings demonstrated that both gut microbiota remodeling and NDGA administration significantly attenuated colonic infiltration of myeloperoxidase (MPO)-positive neutrophils in the DSS-induced murine colitis model (Figure S10A-D).

**Figure 5.**
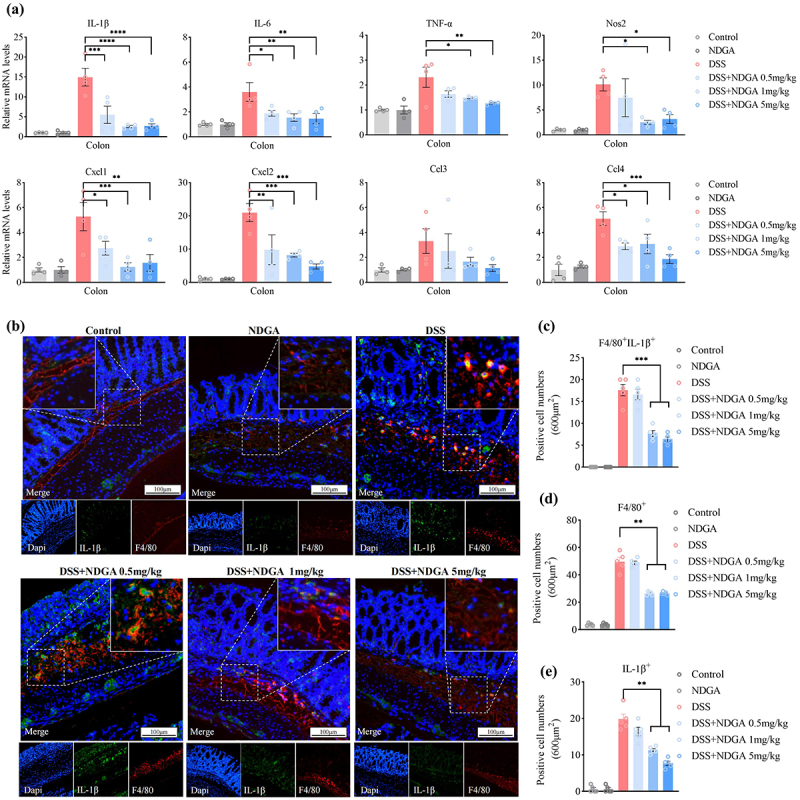
NDGA decreases the accumulation of colonic inflammatory macrophage in the DSS-induced colitis model. (a) qRT-PCR detected pro-inflammatory factor levels in mouse colon tissues; (b) Immunofluorescence detection of F4/80 and IL-1β-positive cells in mouse colon tissues; (c–e) Counts of F4/80-positive, IL-1β-positive, and F4/80 and IL-1β-positive cells.

Macrophages are known to secrete various pro-inflammatory cytokines and chemokines that contribute to the heightened immune response during colitis. The qRT-PCR analysis revealed that NDGA administration significantly downregulated the mRNA expression of multiple macrophage-derived cytokines and chemokines, including IL-6, IL-1β, TNF-α, NOS2, CXCL1/2, and CCL3/4, in the DSS-treated colon (Figure 5(a)). Moreover, NDGA treatment dose-dependently reduced the accumulation of F4/80^+^ and IL-1β^+^ macrophages in the colon (Figure 5(b–e)).

(Data for each group are expressed as mean ± SEM, *n* = 5. *p < 0.05, **p < 0.01, ***p < 0.001; analyzed by one-way ANOVA with Holm-Sidak post hoc tests).

Considering the pivotal role of *Lactobacillus* in NDGA generation, we further evaluated its protective effect on DSS-induced intestinal inflammation in aged mice. Here, we discovered that colonizing aged mice with *L.johnsonii*, *L.murinus*, and *L.reuteri* partially restored fecal NDGA levels (Figure S11A). Besides, colonization by *Lactobacillus* significantly mitigated body weight loss, bloody stools, colon shortening, and histological injury in aged mice following DSS treatment (Figure S11B-H). Additionally, *Lactobacillus* also effectively reduced the infiltration of F4/80^+^ and IL-1β^+^ inflammatory macrophages, confirming inhibition of macrophage pyroptosis (Figure S11I-J). Taken together, these findings suggest that NDGA protects against DSS-induced colitis by suppressing pro-inflammatory macrophage activation.

### NDGA decreases macrophage-released inflammatory mediators upon LPS/ATP stimulation

**Figure 6.**
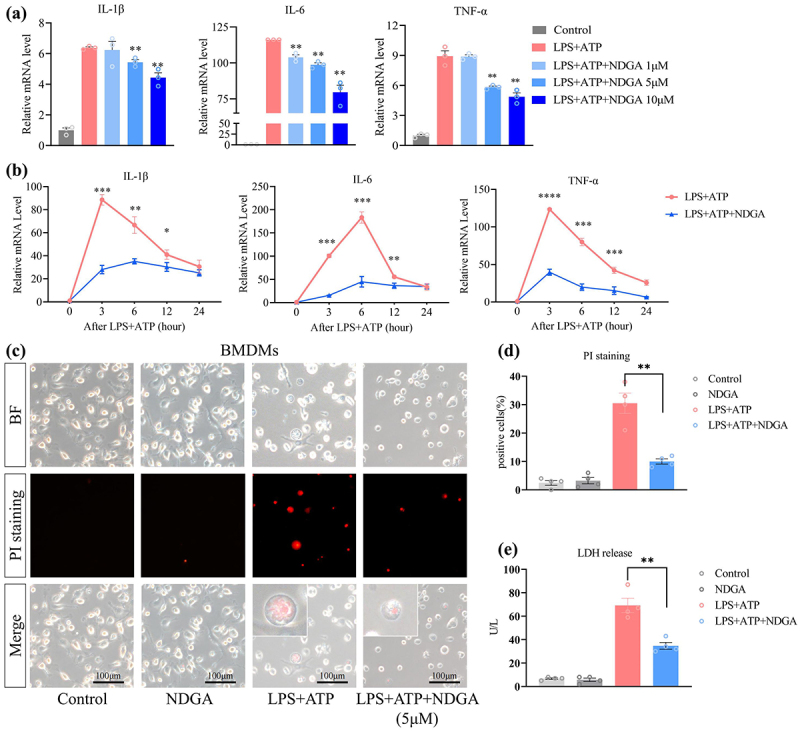
NDGA inhibits LPS/ATP-induced macrophage pyroptosis in BMDMs. (a) qRT-PCR detected pro-inflammatory factor levels in BMDMs treated with NDGA at the indicated dosage; (b) qRT-PCR detected pro-inflammatory factor levels in BMDMs treated with NDGA (5μM) at the indicated time; (c–d) PI staining of BMDMs and the quantitative analysis of positive cells; (e) levels of LDH released from the supernatants of BMDMs.

The precise anti-inflammatory mechanisms of NDGA in inflammatory macrophages remain unclear. To investigate the direct inhibitory effects of NDGA on macrophage activation, we isolated BMDMs and subjected them to in vitro analysis. NDGA treatment dose- and time-dependently suppressed the expression of multiple inflammatory mediators, including IL-1β, IL-6, and TNF-α in lipopolysaccharide (LPS)/adenosine triphosphate (ATP)-stimulated BMDMs (Figure 6(a–b)). Furthermore, NDGA attenuated LPS/ATP-induced pyroptotic cell death, as evidenced by a reduction in propidium iodide (PI)-positive cells (Figure 6(c–d)). Similarly, NDGA decreased the release of lactate dehydrogenase (LDH) from LPS/ATP-stimulated BMDMs (Figure 6(e)). These findings provide compelling evidence that NDGA directly inhibits the secretion of inflammatory mediators by macrophages. Consistently, the anti-inflammatory effects of NDGA were observed in the RAW264.7 macrophage cell line (Figure S12A-B).

(Data for each group are expressed as mean ± SEM, *n* = 3-4. *p < 0.05, **p < 0.01, ***p < 0.001; analyzed by one-way ANOVA with Holm-Sidak post hoc tests).

### NDGA restricts macrophage-released inflammatory mediators via regulating GSDMD/NR4A1/NLRP3 signaling

**Figure 7.**
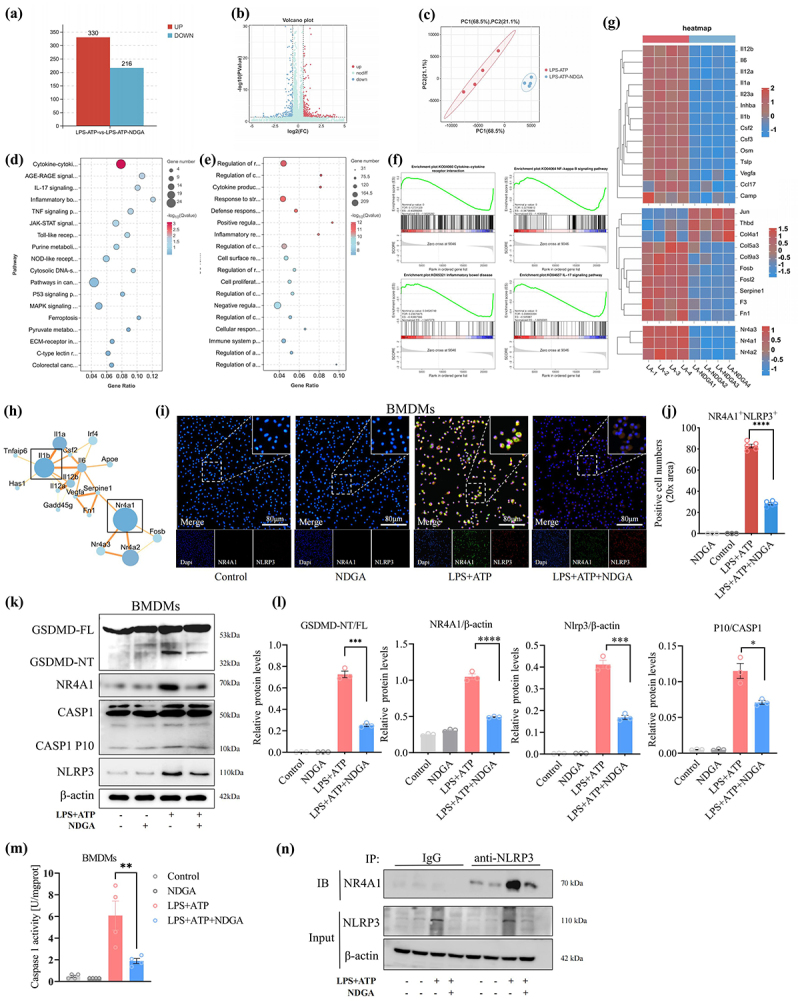
NDGA modulates the NR4A1-NLRP3 axis in pyroptotic macrophages. (a) Histogram of the number of differential genes between the two groups of BMDMs (*n* = 4); (b) volcano plot of differential gene expression; (c) principle coordination analysis between the two groups of BMDMs; (d–e) KEGG and GO signaling pathway enrichment analyses of the differential genes in BMDMs; (f) GSEA analyses demonstrating the pathways inhibited by NDGA;(g) heatmap showing the differential gene expression in two groups of BMDMs;(h) PPI analyses of the differential genes in BMDMs; (i–j) immunofluorescent co-localization assay of NLRP3 and NR4A1 in BMDMs and their quantitative analysis. (k–l) immunoblot detection of GSDMD, NR4A1, NLRP3 and Caspase1 protein levels in BMDMs and their quantitative analysis; (m) the caspase-1 activity in BMDMs detected using a specific detection kit; (n) Co-IP analysis of the protein interaction between NR4A1 and NLRP3 in BMDMs.

To elucidate the molecular mechanisms underlying NDGA’s inhibitory effects on macrophage pyroptosis, we performed RNA-sequencing analysis on LPS/ATP-stimulated BMDMs treated with or without NDGA. This analysis identified 546 differentially expressed genes in LPS/ATP-stimulated BMDMs (Figure 7(a-b)). Principal component analysis (PCA) demonstrated a clear separation between NDGA-treated and untreated groups, indicating a consistent effect of NDGA on the transcriptional profile of LPS/ATP-stimulated macrophages (Figure 7(c)). Kyoto Encyclopedia of Genes and Genomes (KEGG) pathway enrichment analysis revealed that NDGA regulates several inflammatory signaling pathways, including cytokine-cytokine receptor interaction, NF-κB signaling, and IBD pathways. Gene Ontology (GO) and Gene Set Enrichment Analysis (GSEA) further confirmed that NDGA modulates cytokine production and inflammatory responses (Figure 7(d-f)). Transcriptomic analysis validated the downregulation of several pro-inflammatory cytokines, including IL-6, IL-1α, IL-1β, and OSM, as well as orphan nuclear receptors NR4A1, NR4A2, and NR4A3 (Figure 7(g)). Protein-protein interaction (PPI) network analysis suggested that the decreased expression of pro-inflammatory cytokines is closely associated with reduced levels of NR4A1 (Figure 7(h)).

(Data for each group are expressed as mean ± SEM, *n* = 3-4. *p < 0.05, **p < 0.01, ***p < 0.001, ****p < 0.0001; analyzed by one-way ANOVA with Holm-Sidak post hoc tests).

IL-1β is a key mediator of the inflammatory response downstream of NLRP3 inflammasome activation and GSDMD-mediated pyroptosis in macrophages.^36,37^ The orphan nuclear receptor NR4A1 is activated by GSDMD-mediated mitochondrial DNA release. Previous studies have demonstrated a positive crosstalk between NR4A1 and NLRP3, which exacerbates pyroptosis and the release of pro-inflammatory cytokines.^38^ Our *in vitro* study further substantiated that NDGA treatment could effectively inhibit LPS/ATP-induced NR4A1 and NLRP3 co-location in BMDMs (Figure 7(i-J)). We next examined NR4A1 and NLRP3 activation in intestinal tissue sections from patients with ulcerative colitis and found increased activation of these proteins in CD68^+^ macrophages in active UC compared to remission (Figure S13A-F). To further investigate the role of NR4A1 in macrophage pyroptosis, BMDMs were treated with Cytosporone B, a specific NR4A1 agonist, prior to LPS/ATP stimulation. Notably, NR4A1 activation induced excessive pyroptotic cell death, as evidenced by an increased abundance of PI-positive cells and elevated LDH release (Figure S14A-C). In LPS/ATP-primed BMDMs, the addition of Cytosporone B increased the expression of several cytokines, including IL-6, TNF-α, and IL-1β (Figure S14D).

Furthermore, LPS/ATP stimulation enhanced GSDMD pore formation and NLRP3 inflammasome activation, as evidenced by increased protein expression of cleaved-caspase-1, GSDMD-NT, NR4A1, and NLRP3. Notably, NDGA treatment significantly attenuated the expression of these proteins (Figure 7(k-l)). Besides, the specific caspase-1 activity detection kit demonstrated that NDGA effectively reduced caspase-1 activity in LPS/ATP-stimulated BMDMs (Figure 7(m)). Next, the results of the co-immunoprecipitation (Co-IP) revealed that NDGA disrupts the NR4A1-NLRP3 protein interaction in LPS/ATP-primed macrophages (Figure 7(n)). These findings suggest that the modulation of the GSDMD/NR4A1/NLRP3 signaling axis may underlie the anti-inflammatory effects of NDGA.

### NDGA ameliorates colitis by inhibiting GSDMD-dependent pyroptosis

**Figure 8.**
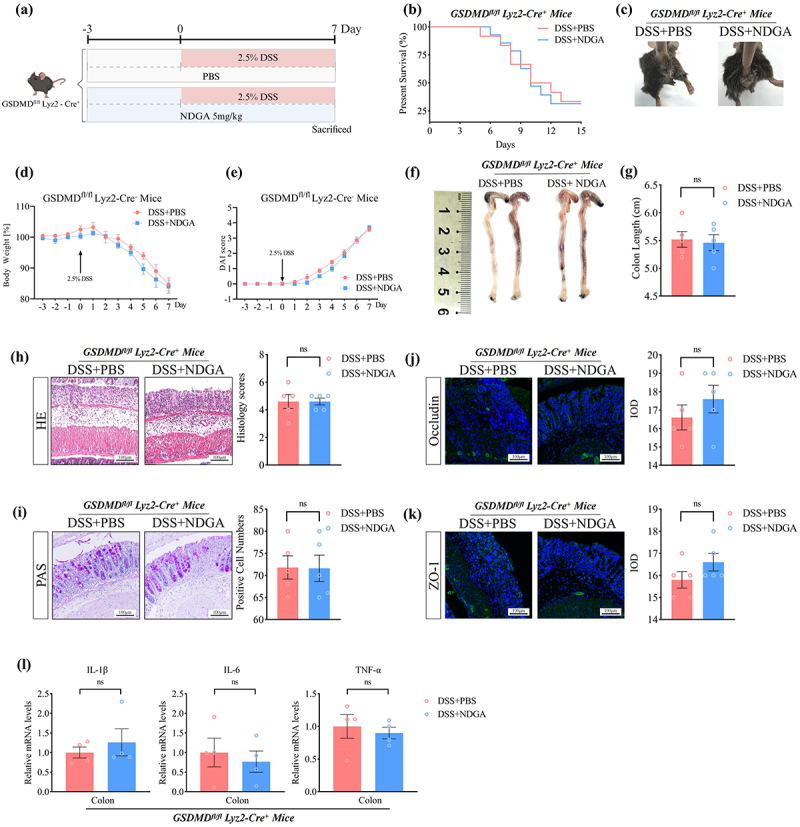
NDGA protects against DSS-induced colitis in a GSDMD-dependent manner. (a) Experimental design of NDGA treatment in GSDMD^fl/fl^Lyz2-Cre mice; (b) The overall survival curves of DSS-treated mice; (c) Bloody stools of DSS-treated mice; (d) Body weight changes were daily monitored after DSS administration;(e) DAI was measured in DSS-treated mice; (f–g) mice were euthanized on day 7, and colon lengths were measured. (h) representative images of the histological-examined colon sections and the pathological scores were quantified. (i) representative images of PAS staining in the DSS-treated colon and its quantitative analysis. (j–k) representative immunofluorescence images of tight junction proteins (occludin, ZO-1) in the DSS-treated colonic tissues and its quantitative analysis.

GSDMD cleavage is essential for inducing NR4A1 activation by forming pores in the mitochondrial membrane, allowing mitochondrial DNA release.^38^ Similarly, NDGA treatment effectively inhibits GSDMD cleavage in the DSS-treated colon (Figure S15A-B). To further investigate the role of GSDMD in NDGA-mediated protection against colitis, we utilized GSDMD^*fl/fl*^Lyz2-Cre mice (Figure 8(a)). Deletion of GSDMD in macrophages significantly attenuated the therapeutic efficacy of NDGA on DSS-induced colitis, as evidenced by the absence of significant differences in survival rates, weight loss, colon shortening, and histological scores between the DSS+PBS and DSS+NDGA groups (Figure 8(b-h)). Additionally, the differential expression of tight junction proteins, mucus layer components, and inflammatory markers was significantly abolished by GSDMD deletion in macrophages (Figure 8(i-l)). Collectively, these findings indicate that NDGA ameliorates colitis development by inhibiting GSDMD/NR4A1/NLRP3-dependent inflammatory macrophage activation.

(Data for each group are expressed as mean ± SEM, *n* = 4-5. n.s, non-significant; analyzed by two-tailed Students’ *t* -test).

## Discussion

**Figure 9.**
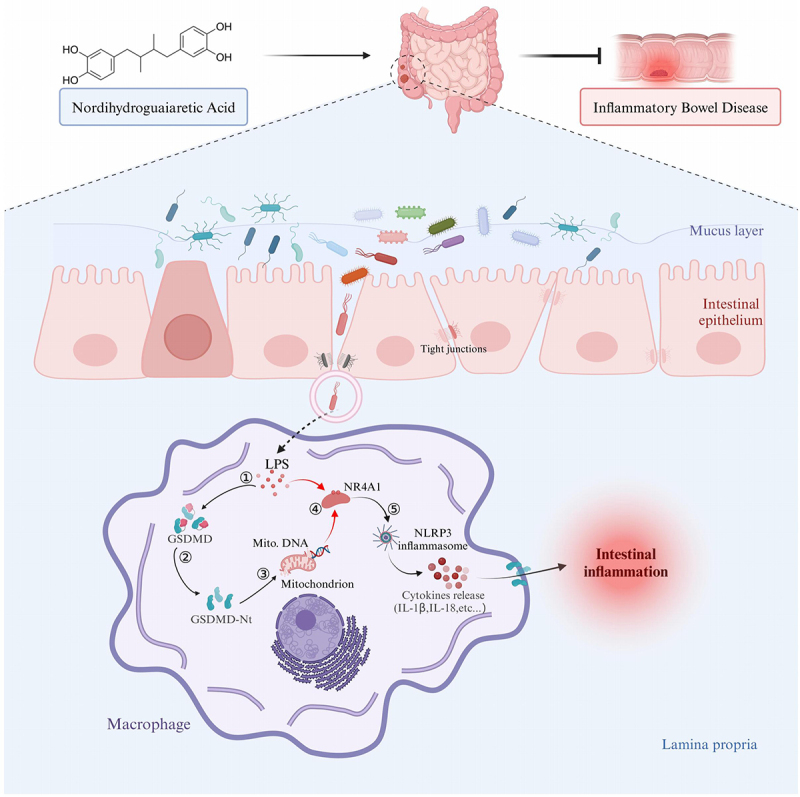
Gut microbial NDGA suppresses inflammatory macrophage activation to regulate epithelial homeostasis and inflammation.This study demonstrates that age-associated dysbiosis leads to reduced NDGA production, resulting in the activation of inflammatory macrophages during colitis development. Conversely, NDGA intervention ameliorates intestinal inflammation and improves barrier function by inhibiting GSDMD/NR4A1/NLRP3-mediated pyroptotic cell death and cytokine release in macrophages.

This study aimed to investigate the impact of age-related dysbiosis on the severity of intestinal inflammation in IBD. We identified NDGA, a metabolite produced by the gut microbiota of young, inflammation-resistant mice, as a protective factor against DSS-induced immune response and barrier dysfunction. Figure 9 summarizes the major mechanisms underlying the beneficial effects of NDGA. By inhibiting GSDMD-mediated pyroptosis in macrophages, NDGA suppresses the GSDMD/NR4A1/NLRP3 signaling axis, thereby regulating epithelial homeostasis and inflammation. Our findings support the concept of gut microbiota aging in the context of IBD. Notably, NDGA may represent a promising therapeutic strategy for IBD.

The prevalence of IBD in the elderly population is steadily increasing, paralleling the global rise in both aging populations and IBD incidence.^5,39^ The gut microbiota undergoes significant changes with aging, which can influence disease outcomes.^15^ While it is well-established that aged mice exhibit increased susceptibility to colitis, the underlying mechanisms remain elusive. The role of age-related dysbiosis in promoting inflammatory disorders in elderly IBD patients remains unclear. In this study, young mice that received gut microbiota from aged mice exhibited increased severity of DSS-induced colitis compared to those receiving microbiota from young mice, suggesting a potential pro-inflammatory role of age-associated dysbiosis in the elderly. Conversely, fecal microbiota remodeling protected aged mice from intestinal inflammation, indicating that the loss of specific core microbiota during aging is associated with immuno-inflammatory dysregulation.

Gut microbiota-derived metabolites continuously modulate immune system development and immune responses, although only a few of these microbial metabolites have been identified and functionally characterized.^21^ Our findings underscore the critical role of microbial-derived NDGA in bridging age-associated dysbiosis and macrophage pyroptosis, thereby modulating colitis progression.^40^ Specifically, we observed that aging-driven depletion of *Lactobacillus* species correlates with diminished fecal NDGA levels, a phenomenon mirrored in both elderly individuals and IBD patients. Restoring NDGA levels, either via microbiota-targeted interventions or direct supplementation, might emerges as a novel strategy to ameliorate IBD colitis.

Gut microbiota plays a critical role in metabolizing dietary phenolics into biologically active compounds through enzymatic action. NDGA, a phenolic derivative of *Larrea tridentata* leaves, has been widely used by Native Americans to treat autoimmune diseases.^41,42^ Previous studies have shown a decrease in fecal NDGA levels in mice fed a high-fat diet, suggesting dietary and microbiota-linked regulation of its production.^34^ However, the role of gut microbiota in NDGA production remains unclear. In this study, we observed a positive correlation between fecal NDGA levels and *Lactobacillus* abundance. This suggests that *Lactobacillus* may play a role in NDGA production, as both NDGA and *Lactobacillus* are highly abundant in young mice. Furthermore, antibiotic treatment significantly reduced fecal NDGA levels in young mice, indicating that commensal gut microbiota is a major source of fecal NDGA. This aligns with broader mechanisms of polyphenol metabolism by gut bacteria, where enzymatic processes like deglycosylation and reduction convert dietary phenolics (e.g., flavonoids, lignans) into bioactive compounds.^20^ NDGA, as a phenolic compound, are originate from plant-based polyphenols and rely on microbial activity for its biotransformation, with *Lactobacillus* potentially contributing to its generation or stability.^42,43^

However, NDGA was undetectable in the cultured supernatant of *Lactobacillus*, with or without dietary polyphenols supplementation. Conversely, mice fed dietary polyphenols showed an increased tendency for fecal NDGA accumulation, indicating that the gut microbiota might mediate the conversion of microbial secondary metabolites to produce NDGA. Such microbial metabolic activity could involve enzymatic conversion of polyphenolic compounds, which warrants further mechanistic investigation.

The interaction between dietary polyphenols and gut microbiota generates bioactive metabolites with significant immunomodulatory properties, as exemplified by desaminotyrosine (DAT) and phloroglucinol. Phloroglucinol tempers macrophage responses to pathogens, reducing pro-inflammatory cytokine production via aryl hydrocarbon receptor (AhR)-mediated signaling.^16^ Similarly, DAT demonstrates anti-inflammatory efficacy in metabolic disorders, attenuating high-fat diet-induced obesity and DSS-induced colitis by maintaining mucosal barrier integrity and reducing systemic inflammation through type I interferon (IFN)-dependent mechanisms.^17^ These findings emphasize phenolic microbial metabolites from dietary sources act as critical mediators of immune homeostasis. In this study, we investigated the therapeutic efficacy of NDGA in the colitis model. Our data demonstrated that NDGA not only reduced the number of NLRP3^+^ and IL-1β^+^ infiltrated macrophages by suppressing pyroptosis but also enhanced intestinal barrier integrity, thereby promoting inflammation resolution in IBD mice.

Conversely, decreased levels of the microbial anti-aging compound NDGA, observed in both elderly individuals and patients with IBD, may contribute to the exacerbation and persistence of chronic inflammation, ultimately accelerating the progression of colitis. While higher levels of NDGA have been observed in the feces of some healthy individuals, a subset of individuals exhibit near-undetectable levels of NDGA, suggesting that the concentration of NDGA is significantly influenced by dietary and environmental factors. Further in-depth studies, utilizing larger cohorts of individuals, are warranted to identify these specific factors.

Chronic inflammation is a hallmark of aging and can contribute to the development of immune-related diseases.^44,45^ To investigate the anti-inflammatory mechanisms of NDGA, we embarked on a study. Pyroptosis is recognized for its role in enhancing the secretion of inflammatory cytokines and DAMPs, thereby facilitating the recruitment of inflammatory cells and contributing to immune-mediated diseases.^46–48^ Notably, pro-inflammatory cytokines secreted by macrophages are highly expressed in the colon of DSS-treated mice but are significantly reduced by therapeutic administration of NDGA.

NR4A1, a pivotal nuclear receptor subfamily 4 group A member, acts as a dual-functional transcriptional regulator in response to extracellular stress.^49,50^ A recent study suggested that NR4A1 becomes activated by detrimental stimuli or mitochondrial DNA, triggering NLRP3 inflammasome activation and pyroptosis in macrophages.^38^ However, the potential interplay between GSDMD and NR4A1 and their collective influence on the inflammatory cascade in IBD remains undetermined. Our study highlights the detrimental role of NR4A1, which is effectively inhibited by NDGA through direct negative regulation. This modulation contributes to the inhibition of macrophage pyroptosis, thereby impeding colitis progression. RNA-seq analysis revealed that NDGA significantly inhibits the expression of NR4A1, a nuclear hormone receptor known to modulate the innate immune response.^49^ A recent study suggested that NR4A1 could directly activate the NLRP3 inflammasome to promote GSDMD-mediated pyroptosis.^38^ In the present study, NDGA effectively diminished the expression levels of NLRP3 and NR4A1 in pyroptotic macrophages, as well as the protein expression levels of cleaved-caspase-1, NLRP3, and GSDMD-NT. Furthermore, activation of murine NR4A1 by a specific agonist induced pyroptotic cell death in BMDMs, accompanied by enhanced cytokine expression. Further studies are required to elucidate the precise regulatory mechanism of NDGA on NR4A1 activation. Additionally, PPI analysis revealed that Serpine1, a key pro-inflammatory mediator that exacerbates IBD-associated intestinal inflammation and fibrosis, is closely associated with the expression of several cytokines. Exploring NDGA’s ability to modulate Serpine1 activation represents a promising research direction. Macrophages are key innate immune cells that play a central role in maintaining tissue homeostasis and orchestrating immune responses. In this study, we focused on NDGA’s effect on macrophages due to their pivotal role in pyroptosis-driven inflammation. Future studies are needed to explore the effect of NDGA on the modulation of other immune cells, such as T cells and neutrophils, which might help provide a more comprehensive understanding of NDGA’s immune modulation mechanisms.

## Conclusion

In summary, by identifying NDGA, a microbial metabolite with anti-pyroptotic properties, our study highlights the crucial role of the gut microbiota in regulating epithelial homeostasis and inflammation. We have uncovered a novel mechanism by which gut microbiota-derived NDGA modulates GSDMD/NR4A1/NLRP3-mediated pyroptosis in macrophages. These findings advance our understanding of “microbiome aging” in the context of colitis development and suggest a potential therapeutic approach for IBD.

## Supplementary Material

Supplemental Material

## Data Availability

The raw metagenome sequencing data of 16S rDNA, single-cell RNA-seq and RNA-seq reported in this paper have been deposited in the Genome Sequence Archive database (accession no. CRA019312, CRA019269 and CRA019267). The raw data of Non-Targeted Metabolomics reported in this paper have been deposited in the OMIX database (accession no. OMIX007519).

## Abbreviations

IBD: Inflammatory Bowel Disease
CD: Crohn’s disease
UC: ulcerative Colitis
FMT: fecal microbiota transplantation
DSS: Dextran Sulfate Sodium
NDGA: Nordihydroguaiaretic acid
H&E: hematoxylin and eosin
PAS: Periodic Acid-Schiff
MUC2: mucin 2
ScRNA-seq: Single-cell RNA sequencing
UMAP: unbiased, graph-based clustering
MRC1: mannose receptor C-type 1
KEGG: Kyoto Encyclopedia of Genes and Genomes
GO: gene ontology
GSEA: gene set enrichment analysis
PI: Propidium iodide
LDH: lactate dehydrogenase
BMDMs: Bone marrow derived macrophages
LPS: lipopolysaccharide
ATP: adenosine triphosphate
GSDMD: Gasdermin D
PPI: protein-protein interaction
NR4A1: nuclear receptor subfamily 4, group A, member 1
NLRP3: NOD-like receptor protein 3
IL: Interleukin
CXCL: C-X-C motif chemokine ligand
TNF-α: Tumor Necrosis Factor α
NOS2: Nitric Oxide Synthase 2
DAMPs: damage-associated molecular patterns
MMP-9: Matrix metalloproteinase-9
LCN2: lipocalin 2
LRG1: leucine-rich alpha-2 glycoprotein 1
Chi3l1: Chitinase 3-like 1
LC-MS: liquid chromatography-mass spectrometry
HPLC: high-performance liquid chromatography
IOD: Integrated optical density

## References

[1] Abraham C, Cho JH. Inflammatory bowel disease. N Engl J Med. 2009;361(21):2066–23. doi: 10.1056/NEJMra0804647. 19923578 PMC3491806

[2] Khor B, Gardet A, Xavier RJ. Genetics and pathogenesis of inflammatory bowel disease. Nature. 2011;474(7351):307–317. doi: 10.1038/nature10209. 21677747 PMC3204665

[3] Singh S, Boland BS, Jess T, Moore AA. Management of inflammatory bowel diseases in older adults. Lancet Gastroenterol Hepatol. 2023;8(4):368–382. doi: 10.1016/S2468-1253(22)00358-2. 36669515

[4] Ruel J, Ruane D, Mehandru S, Gower-Rousseau C, Colombel JF. Ibd across the age spectrum: is it the same disease? Nat Rev Gastroenterol Hepatol. 2014;11(2):88–98. doi: 10.1038/nrgastro.2013.240. 24345891

[5] Jeuring SF, van den Heuvel Tr, Zeegers MP, Hameeteman WH, Romberg-Camps MJ, Oostenbrug LE, Masclee AA, Jonkers DM, Pierik MJ, van den Heuvel TRA. Epidemiology and long-term outcome of inflammatory bowel disease diagnosed at elderly age-an increasing distinct entity? Inflamm Bowel Dis. 2016;22(6):1425–1434. doi: 10.1097/MIB.0000000000000738. 26933752

[6] Hong SJ, Katz S. The elderly ibd patient in the modern era: changing paradigms in risk stratification and therapeutic management. Therap Adv Gastroenterol. 2021;14:17562848211023399. doi: 10.1177/17562848211023399. PMC8255562 34276809

[7] Olén O, Askling J, Sachs MC, Neovius M, Smedby KE, Ekbom A, Ludvigsson JF. Mortality in adult-onset and elderly-onset ibd: a nationwide register-based cohort study 1964-2014. Gut. 2020;69(3):453–461. doi: 10.1136/gutjnl-2018-317572. 31092591

[8] Kawamoto S, Hara E. Crosstalk between gut microbiota and cellular senescence: a vicious cycle leading to aging gut. Trends Cell Biol. 2024;34(8):626–635. doi: 10.1016/j.tcb.2023.12.004. 38220548

[9] Ling Z, Liu X, Cheng Y, Yan X, Wu S. Gut microbiota and aging. Crit Rev Food Sci Nutr. 2022;62(13):3509–3534. doi: 10.1080/10408398.2020.1867054. 33377391

[10] Badal VD, Vaccariello ED, Murray ER, Yu KE, Knight R, Jeste DV, Nguyen TT. The gut microbiome, aging, and longevity: a systematic review. Nutrients. 2020;12(12):3759. doi: 10.3390/nu12123759. 33297486 PMC7762384

[11] Adak A, Khan MR. An insight into gut microbiota and its functionalities. Cell Mol Life Sci. 2019;76(3):473–493. doi: 10.1007/s00018-018-2943-4. 30317530 PMC11105460

[12] Thaiss CA, Zmora N, Levy M, Elinav E. The microbiome and innate immunity. Nature. 2016;535(7610):65–74. doi: 10.1038/nature18847. 27383981

[13] Schirmer M, Garner A, Vlamakis H, Xavier RJ. Microbial genes and pathways in inflammatory bowel disease. Nat Rev Microbiol. 2019;17(8):497–511. doi: 10.1038/s41579-019-0213-6. 31249397 PMC6759048

[14] Pu Y, Sun Z, Zhang H, Huang Q, Wang Z, Mei Z, Wang P, Kong M, Yang W, Lin C, et al. Gut microbial features and circulating metabolomic signatures of frailty in older adults. Nat Aging. 2024;4(9):1249–1262. doi: 10.1038/s43587-024-00678-0. 39054372

[15] DeJong EN, Surette MG, Bowdish DME. The gut microbiota and unhealthy aging: disentangling cause from consequence. Cell Host & Microbe. 2020;28(2):180–189. doi: 10.1016/j.chom.2020.07.013. 32791111

[16] Castelo J, Araujo-Aris S, Barriales D, Tanner Pasco S, Seoane I, Peña-Cearra A, Palacios A, Simó C, Garcia-Cañas V, Khamwong M, et al. The microbiota metabolite, phloroglucinol, confers long-term protection against inflammation. Gut Microbes. 2024;16(1):2438829. doi: 10.1080/19490976.2024.2438829. 39676480 PMC11651279

[17] Wei Y, Gao J, Kou Y, Liu M, Meng L, Zheng X, Xu S, Liang M, Sun H, Liu Z, et al. The intestinal microbial metabolite desaminotyrosine is an anti-inflammatory molecule that modulates local and systemic immune homeostasis. FASEB J. 2020;34(12):16117–16128. doi: 10.1096/fj.201902900RR. 33047367

[18] Steed AL, Christophi GP, Kaiko GE, Sun L, Goodwin VM, Jain U, Esaulova E, Artyomov MN, Morales DJ, Holtzman MJ, et al. The microbial metabolite desaminotyrosine protects from influenza through type i interferon. Science. 2017;357(6350):498–502. doi: 10.1126/science.aam5336. 28774928 PMC5753406

[19] Mithul Aravind S, Wichienchot S, Tsao R, Ramakrishnan S, Chakkaravarthi S. Role of dietary polyphenols on gut microbiota, their metabolites and health benefits. Food Res Int. 2021;142:110189. doi: 10.1016/j.foodres.2021.110189. 33773665

[20] Wan MLY, Co VA, El-Nezami H. Dietary polyphenol impact on gut health and microbiota. Crit Rev Food Sci Nutr. 2021;61(4):690–711. doi: 10.1080/10408398.2020.1744512. 32208932

[21] Yang W, Cong Y. Gut microbiota-derived metabolites in the regulation of host immune responses and immune-related inflammatory diseases. Cell Mol Immunol. 2021;18(4):866–877. doi: 10.1038/s41423-021-00661-4. 33707689 PMC8115644

[22] Lavelle A, Sokol H. Gut microbiota-derived metabolites as key actors in inflammatory bowel disease. Nat Rev Gastroenterol Hepatol. 2020;17(4):223–237. doi: 10.1038/s41575-019-0258-z. 32076145

[23] Miao R, Jiang C, Chang WY, Zhang H, An J, Ho F, Chen P, Zhang H, Junqueira C, Amgalan D, et al. Gasdermin d permeabilization of mitochondrial inner and outer membranes accelerates and enhances pyroptosis. Immunity. 2023;56(11):2523–2541.e2528. doi: 10.1016/j.immuni.2023.10.004. 37924812 PMC10872579

[24] Shi J, Gao W, Shao F. Pyroptosis: gasdermin-mediated programmed necrotic cell death. Trends Biochem Sci. 2017;42(4):245–254. doi: 10.1016/j.tibs.2016.10.004. 27932073

[25] Xu J, Núñez G. The nlrp3 inflammasome: activation and regulation. Trends Biochem Sci. 2023;48(4):331–344. doi: 10.1016/j.tibs.2022.10.002. 36336552 PMC10023278

[26] Huang C, Tan H, Song M, Liu K, Liu H, Wang J, Shi Y, Hou F, Zhou Q, Huang R, et al. Maternal western diet mediates susceptibility of offspring to crohn’s-like colitis by deoxycholate generation. Microbiome. 2023;11(1):96. doi: 10.1186/s40168-023-01546-6. 37131223 PMC10155335

[27] Zhao Y, Zhong X, Yan J, Sun C, Zhao X, Wang X. Potential roles of gut microbes in biotransformation of natural products: an overview. Front Microbiol. 2022;13:956378. doi: 10.3389/fmicb.2022.956378. 36246222 PMC9560768

[28] Arifuzzaman M, Collins N, Guo CJ, Artis D. Nutritional regulation of microbiota-derived metabolites: implications for immunity and inflammation. Immunity. 2024;57(1):14–27. doi: 10.1016/j.immuni.2023.12.009. 38198849 PMC10795735

[29] Spindler SR, Mote PL, Lublin AL, Flegal JM, Dhahbi JM, Li R. Nordihydroguaiaretic acid extends the lifespan of drosophila and mice, increases mortality-related tumors and hemorrhagic diathesis, and alters energy homeostasis in mice. J Geron Biol Sci Med Sci. 2015;70(12):1479–1489. doi: 10.1093/gerona/glu190. PMC4631105 25380600

[30] Gong S, Yan Z, Liu Z, Niu M, Fang H, Li N, Huang C, Li L, Chen G, Luo H, et al. Intestinal microbiota mediates the susceptibility to polymicrobial sepsis-induced liver injury by granisetron generation in mice. Hepatology. 2019;69(4):1751–1767. doi: 10.1002/hep.30361. 30506577

[31] Huang C, Wang J, Liu H, Huang R, Yan X, Song M, Tan G, Zhi F. Ketone body β-hydroxybutyrate ameliorates colitis by promoting m2 macrophage polarization through the stat6-dependent signaling pathway. BMC Med. 2022;20(1):148. doi: 10.1186/s12916-022-02352-x. 35422042 PMC9011974

[32] Ha CY, Katz S. Clinical implications of ageing for the management of ibd. Nat Rev Gastroenterol Hepatol. 2014;11(2):128–138. doi: 10.1038/nrgastro.2013.241. 24345890

[33] Miyoshi J, Miyoshi S, Delmont TO, Cham C, Lee STM, Sakatani A, Yang K, Shan Y, Kennedy M, Kiefl E, et al. Early-life microbial restitution reduces colitis risk promoted by antibiotic-induced gut dysbiosis in interleukin 10(-/-) mice. Gastroenterology. 2021;161(3):940–952.e915. doi: 10.1053/j.gastro.2021.05.054. 34111469 PMC8577987

[34] Yang J, Wei H, Zhou Y, Szeto CH, Li C, Lin Y, Coker OO, Lau HCH, Chan AWH, Sung JJY, et al. High-fat diet promotes colorectal tumorigenesis through modulating gut microbiota and metabolites. Gastroenterology. 2022;162(1):135–149.e132. doi: 10.1053/j.gastro.2021.08.041. 34461052

[35] Krishnan S, Ding Y, Saedi N, Choi M, Sridharan GV, Sherr DH, Yarmush ML, Alaniz RC, Jayaraman A, Lee K. Gut microbiota-derived tryptophan metabolites modulate inflammatory response in hepatocytes and macrophages. Cell Rep. 2018;23(4):1099–1111. doi: 10.1016/j.celrep.2018.03.109. 29694888 PMC6392449

[36] Rathinam VAK, Zhao Y, Shao F. Innate immunity to intracellular lps. Nat Immunol. 2019;20(5):527–533. doi: 10.1038/s41590-019-0368-3. 30962589 PMC7668400

[37] Shi J, Zhao Y, Wang K, Shi X, Wang Y, Huang H, Zhuang Y, Cai T, Wang F, Shao F. Cleavage of gsdmd by inflammatory caspases determines pyroptotic cell death. Nature. 2015;526(7575):660–665. doi: 10.1038/nature15514. 26375003

[38] Zhu F, Ma J, Li W, Liu Q, Qin X, Qian Y, Wang C, Zhang Y, Li Y, Jiang D, et al. The orphan receptor nur77 binds cytoplasmic lps to activate the non-canonical nlrp3 inflammasome. Immunity. 2023;56(4):753–767.e758. doi: 10.1016/j.immuni.2023.03.003. 37001519

[39] Ng SC, Shi HY, Hamidi N, Underwood FE, Tang W, Benchimol EI, Panaccione R, Ghosh S, Wu JCY, Chan FKL, et al. Worldwide incidence and prevalence of inflammatory bowel disease in the 21st century: a systematic review of population-based studies. Lancet. 2017;390(10114):2769–2778. doi: 10.1016/S0140-6736(17)32448-0. 29050646

[40] Manda G, Rojo AI, Martínez-Klimova E, Pedraza-Chaverri J, Cuadrado A. Nordihydroguaiaretic acid: from herbal medicine to clinical development for cancer and chronic diseases. Front Pharmacol. 2020;11:151. doi: 10.3389/fphar.2020.00151. 32184727 PMC7058590

[41] Guan X, Zhu S, Song J, Liu K, Liu M, Xie L, Wang Y, Wu J, Xu X, Pang T. Microglial cmpk2 promotes neuroinflammation and brain injury after ischemic stroke. Cell Reports Med. 2024;5(5):101522. doi: 10.1016/j.xcrm.2024.101522. PMC11148565 38701781

[42] Arteaga S, Andrade-Cetto A, Cárdenas R. Larrea tridentata (creosote bush), an abundant plant of Mexican and us-American deserts and its metabolite nordihydroguaiaretic acid. J Ethnopharmacol. 2005;98(3):231–239. doi: 10.1016/j.jep.2005.02.002. 15814253

[43] Muñoz R, Rivas BL, Rodríguez H, Esteban-Torres M, Reverón I, Santamaría L, Landete JM, Plaza-Vinuesa L, Sánchez-Arroyo A, Jiménez N, et al. Food phenolics and lactiplantibacillus plantarum. Int J Food Microbiol. 2024;412:110555. doi: 10.1016/j.ijfoodmicro.2023.110555. 38199014

[44] Li X, Ma L. From biological aging to functional decline: insights into chronic inflammation and intrinsic capacity. Ageing researchrev. 2024;93:102175. doi: 10.1016/j.arr.2023.102175. 38145874

[45] Franceschi C, Garagnani P, Parini P, Giuliani C, Santoro A. Inflammaging: a new immune-metabolic viewpoint for age-related diseases. Nat Rev Endocrinol. 2018;14(10):576–590. doi: 10.1038/s41574-018-0059-4. 30046148

[46] Rao Z, Zhu Y, Yang P, Chen Z, Xia Y, Qiao C, Liu W, Deng H, Li J, Ning P, et al. Pyroptosis in inflammatory diseases and cancer. Theranostics. 2022;12(9):4310–4329. doi: 10.7150/thno.71086. 35673561 PMC9169370

[47] Kovacs SB, Miao EA. Gasdermins: effectors of pyroptosis. Trends Cell Biol. 2017;27(9):673–684. doi: 10.1016/j.tcb.2017.05.005. 28619472 PMC5565696

[48] Yu P, Zhang X, Liu N, Tang L, Peng C, Chen X. Pyroptosis: mechanisms and diseases. Signal Transduct Targeted Ther. 2021;6(1):128. doi: 10.1038/s41392-021-00507-5. PMC8005494 33776057

[49] Liu X, Wang Y, Lu H, Li J, Yan X, Xiao M, Hao J, Alekseev A, Khong H, Chen T, et al. Genome-wide analysis identifies nr4a1 as a key mediator of t cell dysfunction. Nature. 2019;567(7749):525–529. doi: 10.1038/s41586-019-0979-8. 30814730 PMC6507425

[50] Palumbo-Zerr K, Zerr P, Distler A, Fliehr J, Mancuso R, Huang J, Mielenz D, Tomcik M, Fürnrohr BG, Scholtysek C, et al. Orphan nuclear receptor nr4a1 regulates transforming growth factor-β signaling and fibrosis. Nat Med. 2015;21(2):150–158. doi: 10.1038/nm.3777. 25581517

