# Supplementary material for: Gut microbial Nordihydroguaiaretic acid suppresses macrophage pyroptosis to regulate epithelial homeostasis and inflammation
Source: Gut Microbes. 2025 Jul 1;17(1):2518338. doi: 10.1080/19490976.2025.2518338 (PMC12233881; doi:10.1080/19490976.2025.2518338)
Supplement: Supplemental Material [file KGMI_A_2518338_SM1104.docx]

**Gut Microbial Nordihydroguaiaretic Acid Suppresses Macrophage Pyroptosis to Regulate Epithelial Homeostasis and Inflammation**

**Jun Wang^1,2 †^, Huishi Tan^3, †^, Ziwen Ye^4, †^, Senhui Weng^1^, Yanqiang Shi^5^, Jiahui Xu^6^, Hongbin Liu^7^, Jierui Li^8^, Linwen Huang^1^, Luyue Zhai^1^, Huishan Luo^1^, Zelong Lin^1^, Cailing Zhong^1^, Jing Tang^1^, Zezheng Wang^1^, Haiyan Zhang^1,2^ *, Beiping Zhang^1,2^ * , Chongyang Huang^1,2^ *.**

^1^Department of Gastroenterology, The Second Affiliated Hospital of Guangzhou University of Chinese Medicine (Guangdong Provincial Hospital of Chinese Medicine), Guangzhou 510120, China.

^2^Guangdong Provincial Key Laboratory of Chinese Medicine for Prevention and Treatment for Refractory Chronic Diseases; State Key Laboratory of Dampness Syndrome of Chinese Medicine; Guangdong Provincial Key Laboratory of Clinical Research on Traditional Chinese Medicine Syndrome; State Key Laboratory of Traditional Chinese Medicine Syndrome/ Department of Gynecologic Oncology; Guangzhou 510120, China.

^3^Department of Gastroenterology and Hepatology, Guangzhou First People's Hospital, School of Medicine, South China University of Technology, Guangzhou 510180, China.

^4^School of Nursing, Guangdong Pharmaceutical University, Guangzhou 510006, China.

^5^Institute of Dermatology and Venereology, Dermatology Hospital, Southern Medical University, Guangzhou 510091, China.

^6^Department of Gastroenterology, The Second Affiliated Hospital of Guangzhou Medical University, Guangzhou, China.

^7^Guangdong Provincial Key Laboratory of Gastroenterology, Institute of Gastroenterology of Guangdong Province, Department of Gastroenterology, Nanfang Hospital, Southern Medical University, Guangzhou 510515, China.

^8^The First Affiliated Hospital, Faculty of Medical Science, Jinan University, Guangzhou 510630, China;

**^†^**These authors contributed equally to this study.

***Correspondence:** Haiyan Zhang [(zhanghaiyan128@126.com](mailto:(zhanghaiyan128@126.com)), Beiping Zhang ([doctorzbp@163.com](mailto:doctorzbp@163.com)) and Chongyang Huang ([hcyoung16@163.com](mailto:hcyoung16@163.com)), Ph.D, Department of Gastroenterology, The Second Affiliated Hospital of Guangzhou University of Traditional Chinese Medicine, Guangzhou 510120, China.

**Supplementary Materials and methods**

**Immunohistochemistry (IHC) staining**

The colons were excised from euthanized mice, fixed in 4% paraformaldehyde, dehydrated, and embedded in paraffin. Paraffin blocks were sectioned into 4-μm thick tissue sections for further analysis. Primary antibodies were used to perform IHC for MPO (Servicebio, GB120016, 1:1000). The results were observed under a microscope (Olympus, Tokyo, Japan).

**Caspase1 activity**

BMDMs were separated as previous. Using the Bradford Protein Colorimetric Assay Kit (Elabscience, E-BC-K168-M, China) to determine protein concentration. The caspase-1 activity was assayed using the caspase-1 activity assay kit (Elabscience, E-CK-A381, China), following the manufacturer's instructions. The absorbance was measured at a wavelength of 405 nm.

**Dietary polyphenols intervention**

Mice were fed a diet supplemented with polyphenols (Secoisolariciresinol Diglucoside (HY-105008) at 20mg/kg, and Sesamin (HY-N0121) at 20mg/kg, provided by MCE company, China) for a duration of 5 days. Following the intervention period, feces were collected for further analysis.

The *Lactobacillus* culture medium was supplemented with polyphenols (Secoisolariciresinol Diglucoside at 50μM, and Sesamin at 50μM) for a duration of 48 hours.

**Supplementary Figure 1**

**
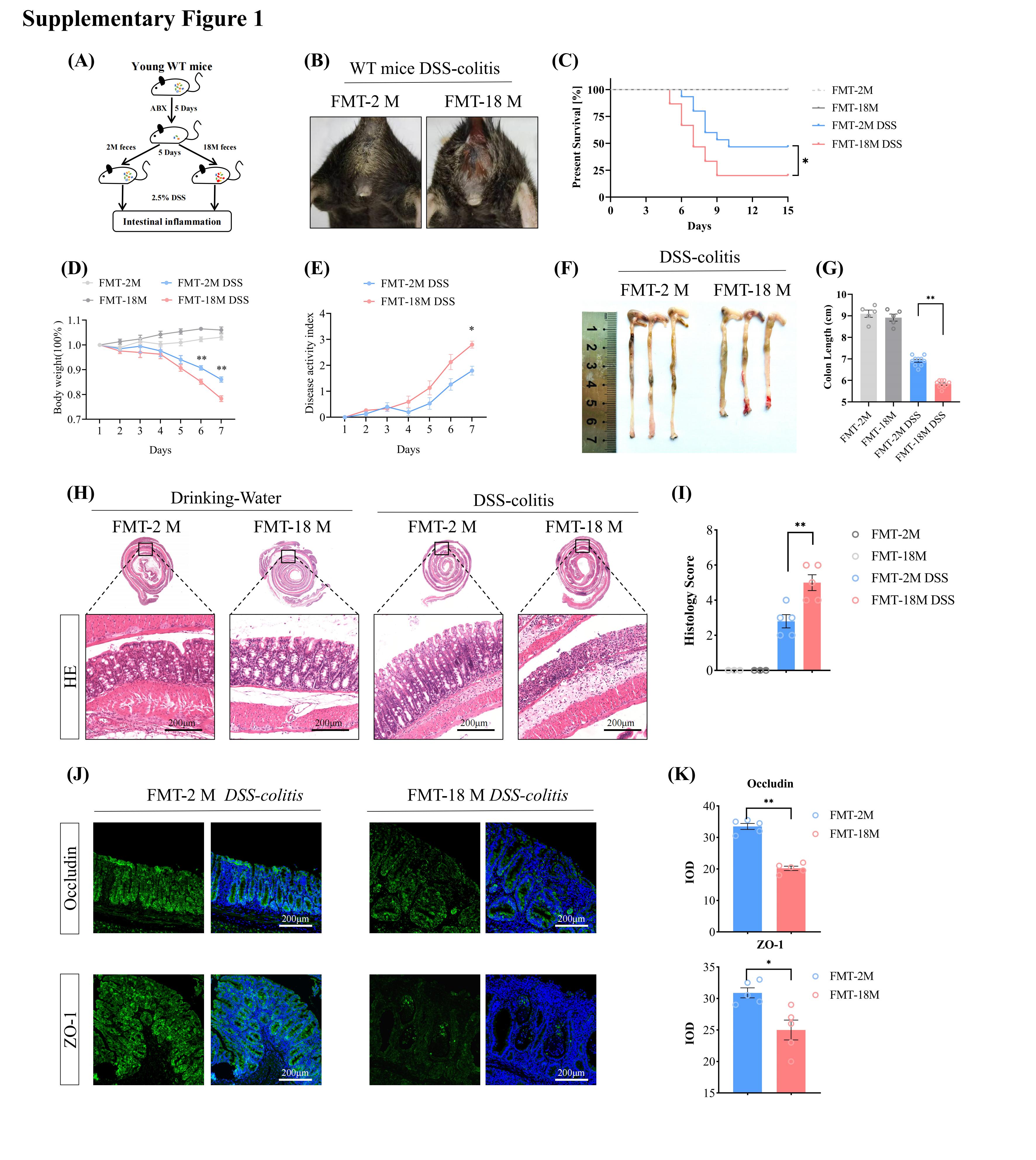
**

**S.Figure 1. Aged-associated dysbiosis aggravates DSS-induced colitis**

(A) Experimental design of fecal bacterial transplantation; (B) Bloody stools of DSS-treated mice; (C) The overall survival curves of DSS-treated mice; (D) Body weight changes were daily monitored after DSS administration; (E) Disease activity index were scored in DSS-treated mice; (F-G) Mice were euthanized on day 7, and colon lengths were measured.; (H-I) Representative images of the histological-examined colon sections and the quantitative analysis; (J-K) Representative Immunofluorescence images of tight junction proteins (Occludin, ZO-1) in the DSS-treated colonic tissues and the quantitative analysis.

(Data in each group were expressed as mean±SEM, n=5. * p <0.05, ** p <0.01; analyzed by one-way ANOVA with Holm-Sidak post hoc tests or two-tailed Students' *t-*test. Data shown are representative of three independent experiments.)

**Supplementary Figure 2**

**
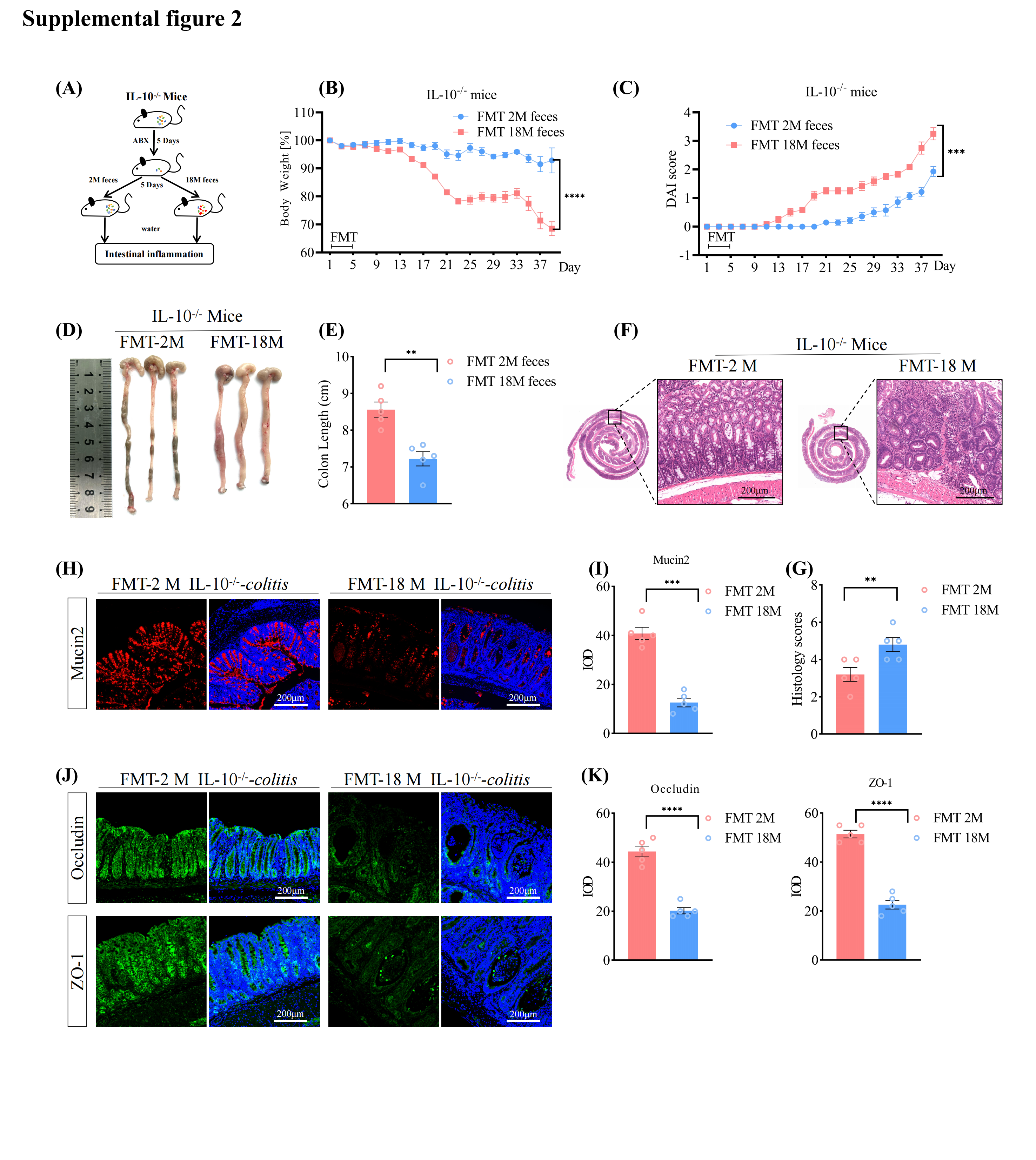
**

**S.Figure 2. Aged-associated dysbiosis derives colitis development in IL-10-deficient mice**

(A) Experimental design of fecal bacterial transplantation; (B) Body weight changes of IL-10-deficient mice were daily monitored; (C) Disease activity index were scored in IL-10-deficient mice; (D-E) Mice were euthanized on day 40, and colon lengths were measured; (F-G) Representative images of the histological-examined colon sections and the quantitative analysis; (H-K) Representative Immunofluorescence images of Mucin2 and tight junction proteins (Occludin, ZO-1) in the colonic tissues and the quantitative analysis.

(Data in each group were expressed as mean±SEM, n=5. ** p <0.01, *** p <0.001, **** p <0.0001; analyzed by one-way ANOVA with Holm-Sidak post hoc tests or two-tailed Students' *t-*test. Data shown are representative of three independent experiments.)

**Supplementary Figure 3**


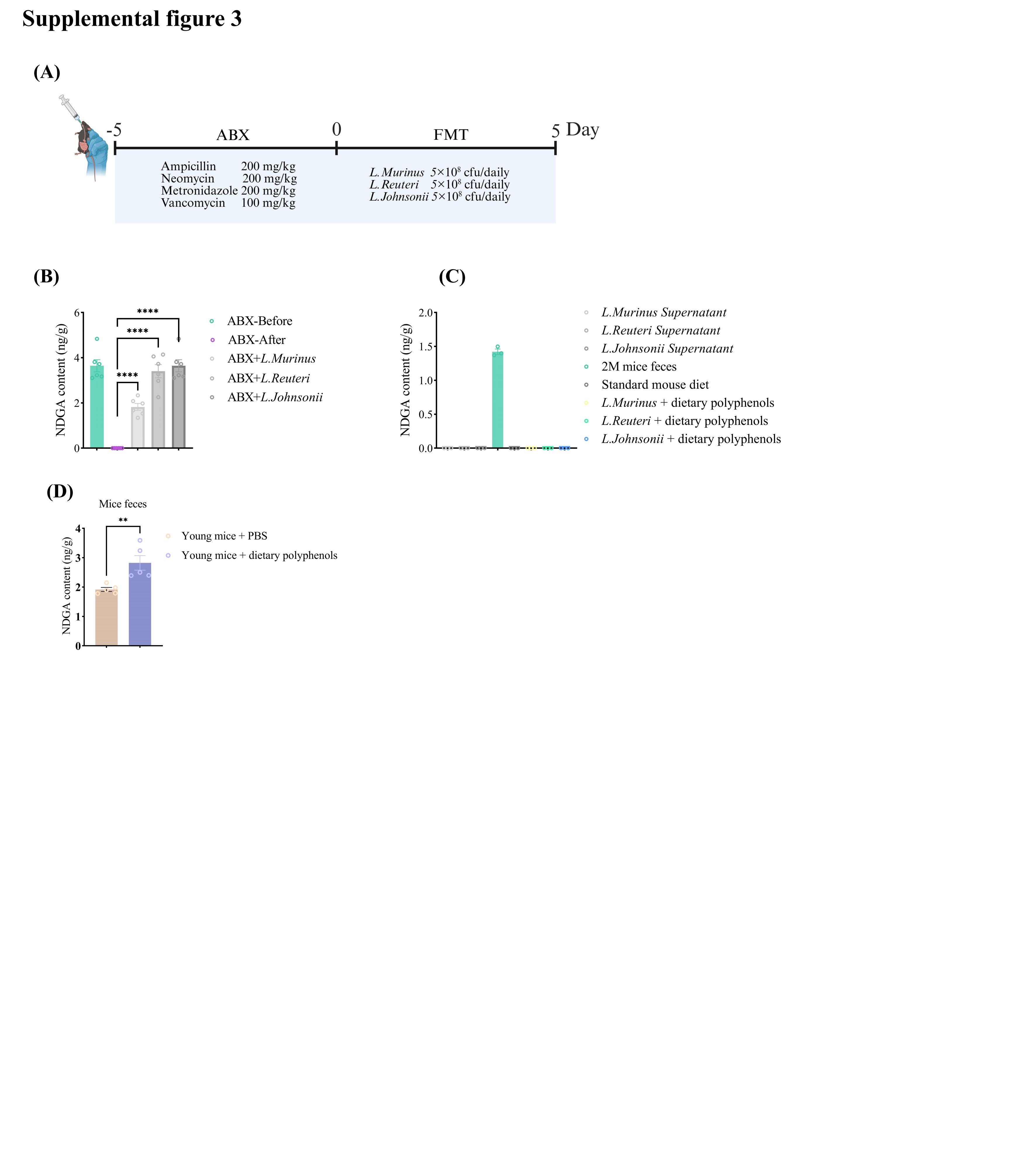


**S.Figure 3. Commensal *Lactobacillus* mediates the generation of intestinal NDGA**

(A) Experimental design of fecal bacterial transplantation; (B) Fecal NDGA levels were detected by metabolomics in mice before and after Lactobacillus colonization; (C) The NDGA levels were detected by metabolomics in *Lactobacillus* supernatant; (D) Fecal NDGA levels were detected by metabolomics in mice with or without dietary polyphenols treatment.

(Data in each group were expressed as mean±SEM, n=3-6. ** p <0.01, **** p <0.0001; analyzed by one-way ANOVA with Holm-Sidak post hoc tests. Data shown are representative of three independent experiments.)

**Supplementary Figure 4**

**
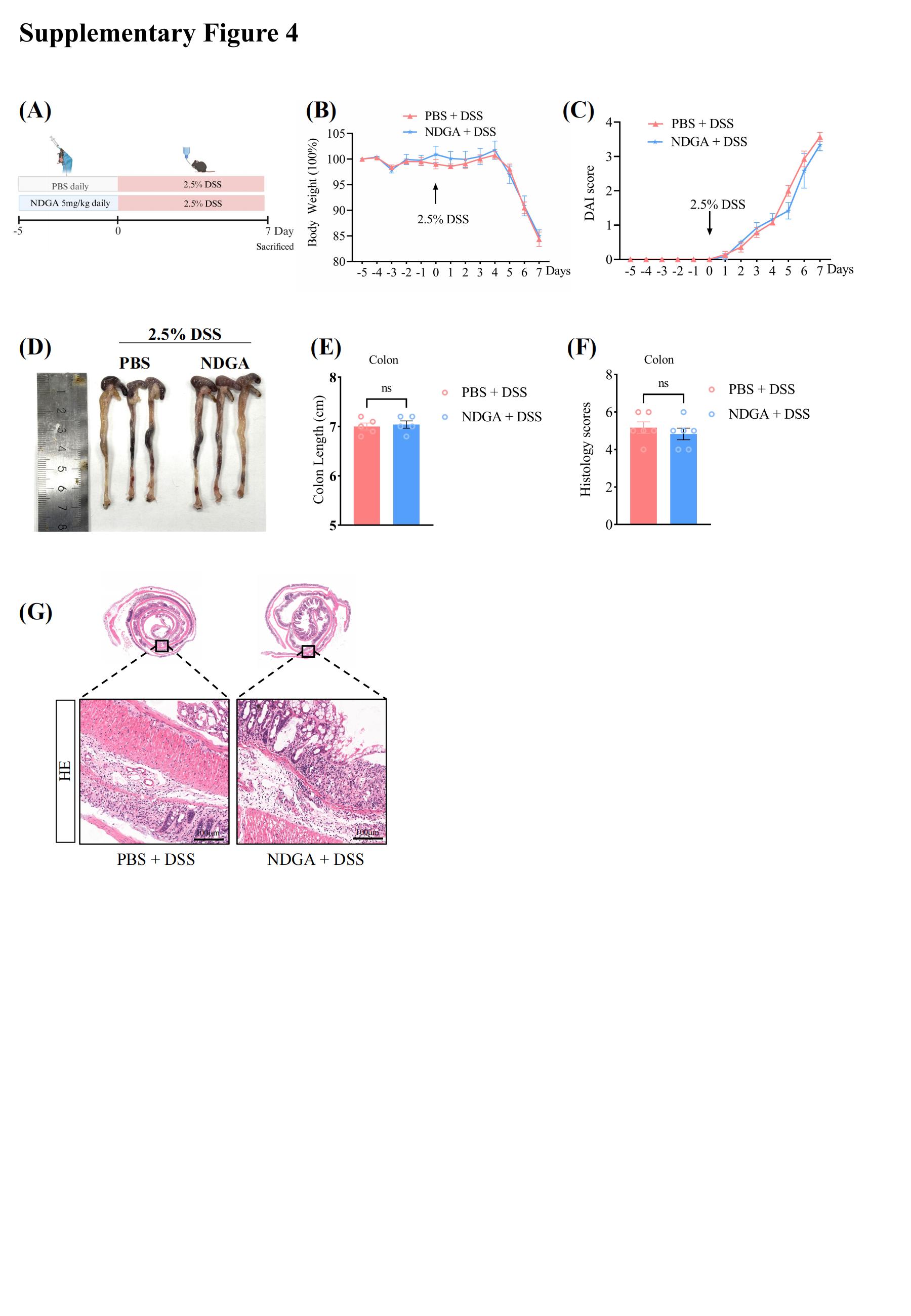
**

**S.Figure 4. NDGA prophylactic administration did not prevent DSS-induced colitis**

(A) Experimental design of NDGA treatment in DSS-induced colitis; (B) Body weight changes were daily monitored after DSS administration; (C) Disease activity index was measured in DSS-treated mice; (D-E) Mice were euthanized on day 7, and colon lengths were measured; (F-G) Representative images of the histologically examined colon sections and the pathological scores were quantified.

(Data for each group are expressed as mean ± SEM, n=5. n.s, non-significant; analyzed by one-way ANOVA with Holm-Sidak post hoc tests. Data shown are representative of three independent experiments.)

**Supplementary Figure 5**

**
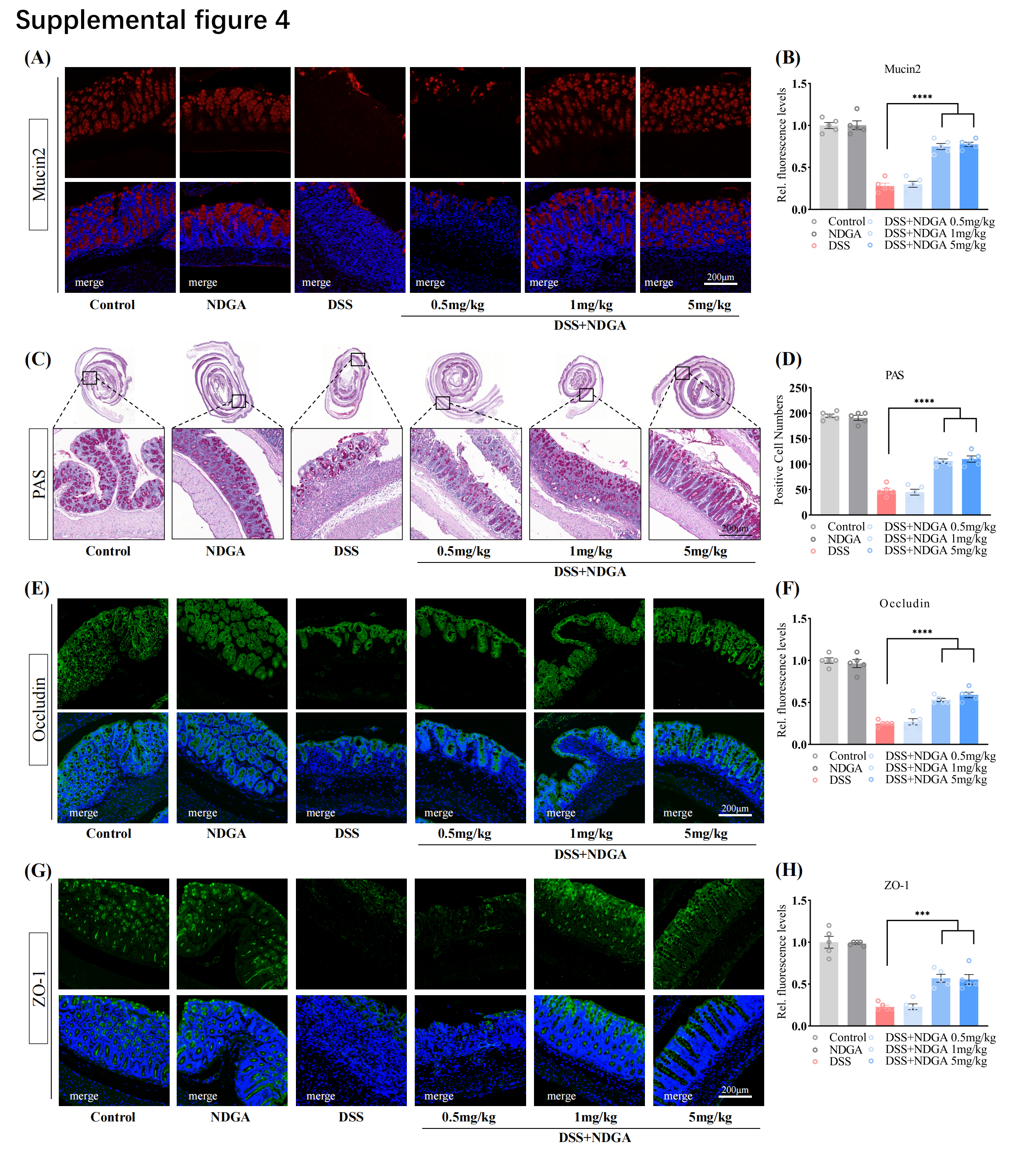
**

**S.Figure 5. NDGA improves intestinal barrier dysfunction in DSS-treated mice**

(A-B) Immunofluorescence staining of MUC2 protein and the quantitative fluorescence analysis of colon tissue; (C-D) PAS staining of mouse colon tissue and the quantitative analysis of positive cells; (E-F) Immunofluorescence labeling of Occludin and the quantitative fluorescence analysis; (G-H) Immunofluorescence labeling of ZO-1 and the quantitative fluorescence analysis.

(Data for each group are expressed as mean ± SEM, n=5. *** p <0.001, **** p <0.0001; analyzed by one-way ANOVA with Holm-Sidak post hoc tests. Data shown are representative of three independent experiments.)

**Supplementary Figure 6**

**
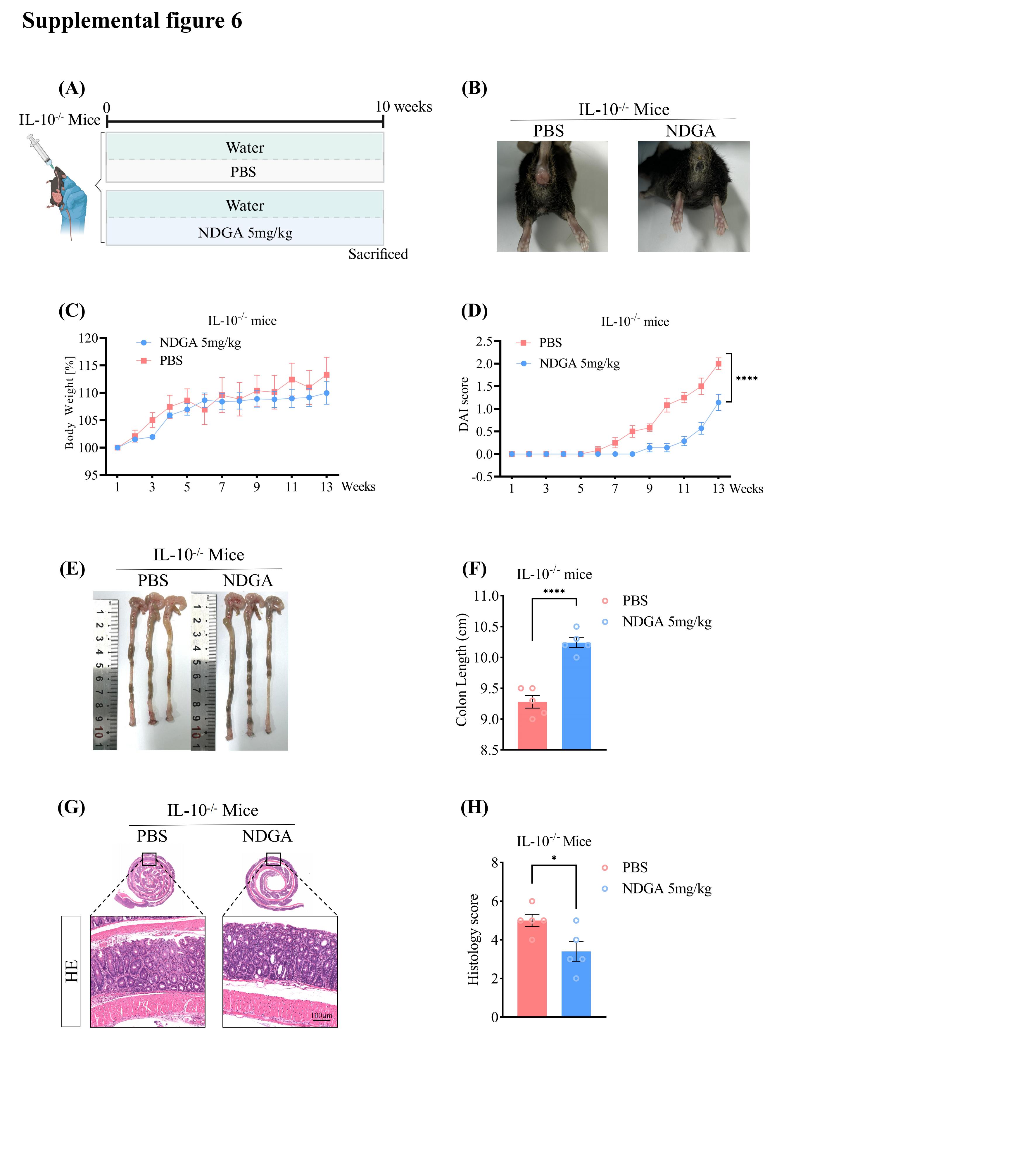
**

**S.Figure 6. NDGA protects against colitis development in IL-10-deficient mice**

(A) Experimental design of NDGA treatment in IL-10-deficient mice; (B) Bloody stools of IL-10-deficient mice; (C-D) Body weight changes and DAI scores were daily monitored after NDGA 5mg/kg administration; (E-F) Mice were euthanized on week 13, and colon lengths were measured; (G-H) Representative images of the histological-examined colon sections and the quantitative analysis.

(Data for each group are expressed as mean ± SEM, n=5. * p <0.05, ** p <0.01, **** p <0.0001; analyzed by two-tailed Student's *t*-test analysis. Data shown are representative of three independent experiments.)

**Supplementary Figure 7**

**
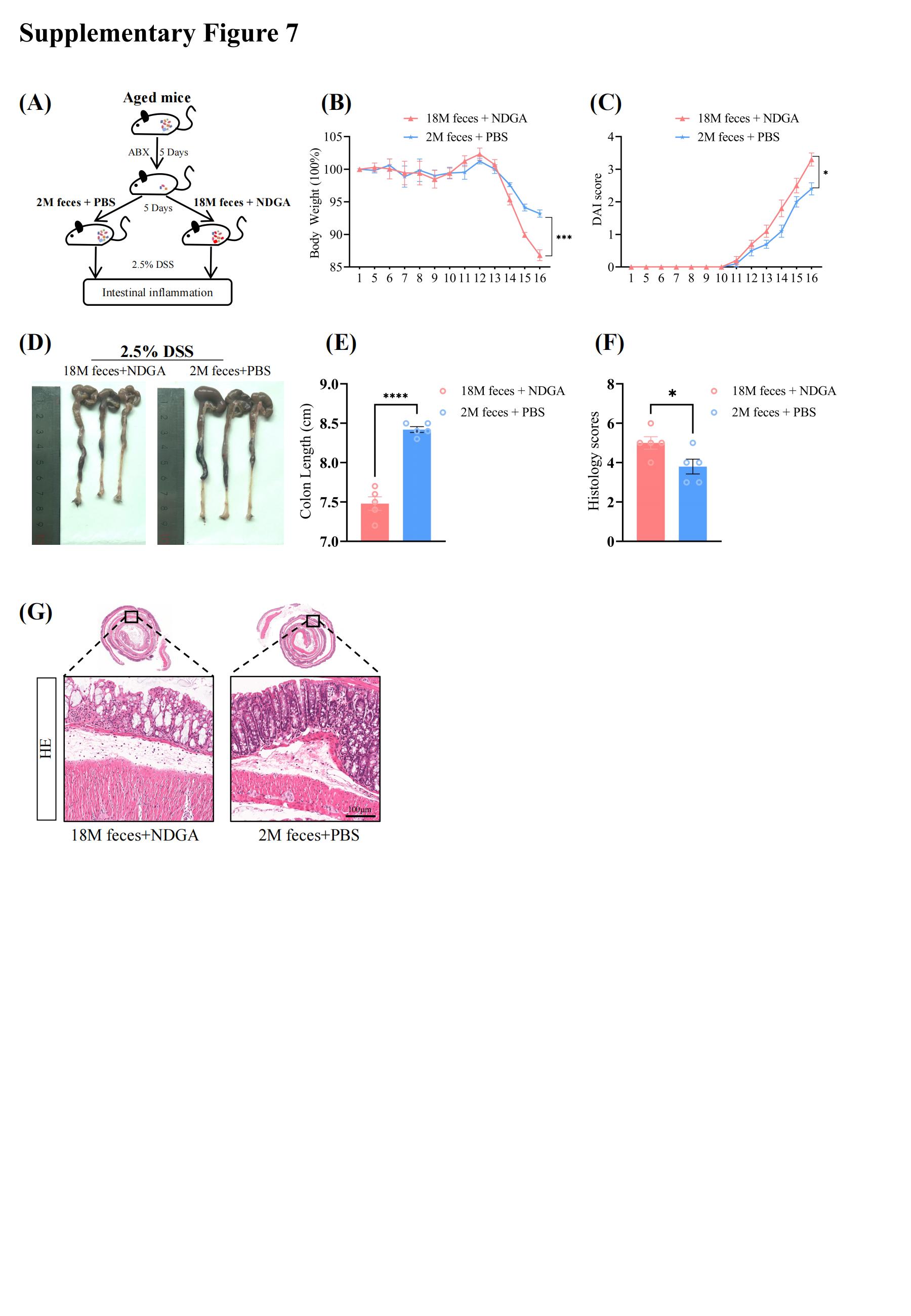
S.Figure 7. NDGA prevent exacerbation of colitis induced by aged microbiota**

(A) Experimental design of NDGA treatment in DSS-induced colitis; (B) Body weight changes were daily monitored after DSS administration; (C) Disease activity index was measured in DSS-treated mice; (D-E) Mice were euthanized on day 7, and colon lengths were measured; (F-G) Representative images of the histological-examined colon sections and the pathological scores were quantified;

(Data for each group are expressed as mean ± SEM, n=5. * p <0.05, ** p <0.01, *** p <0.001, **** p <0.0001; analyzed by one-way ANOVA with Holm-Sidak post hoc tests. Data shown are representative of three independent experiments.)

**Supplementary Figure 8**

**
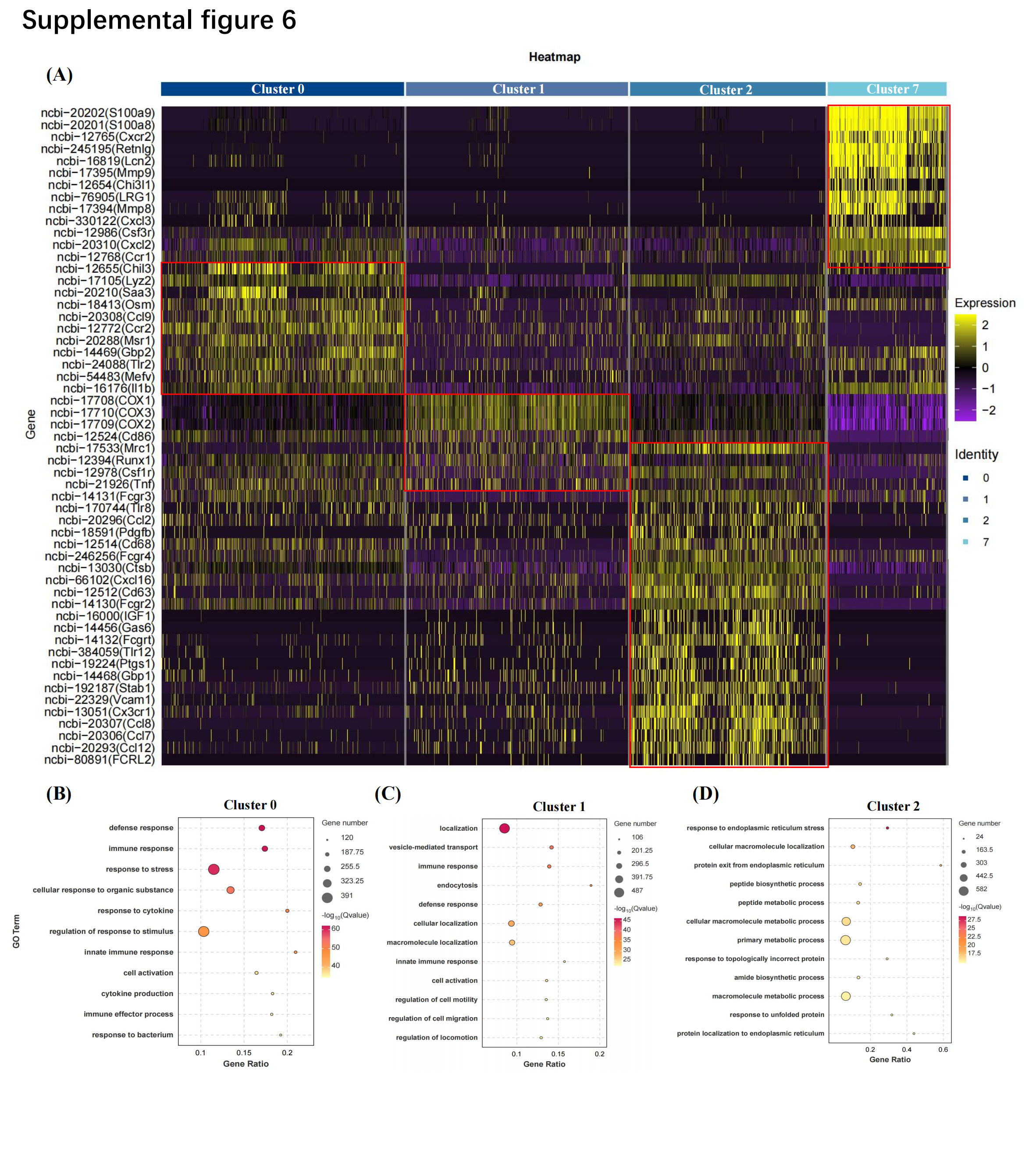
**

**S.Figure 8. Single-cell transcriptome profiling of colon and clustering of cell subsets.**

(A) Heatmap of overall differential gene expression of indicated immune cells in clusters 0, 1, 2, and 7. (B-D) GO pathway enrichment analysis of different macrophage subsets.

**Supplementary Figure 9**

**
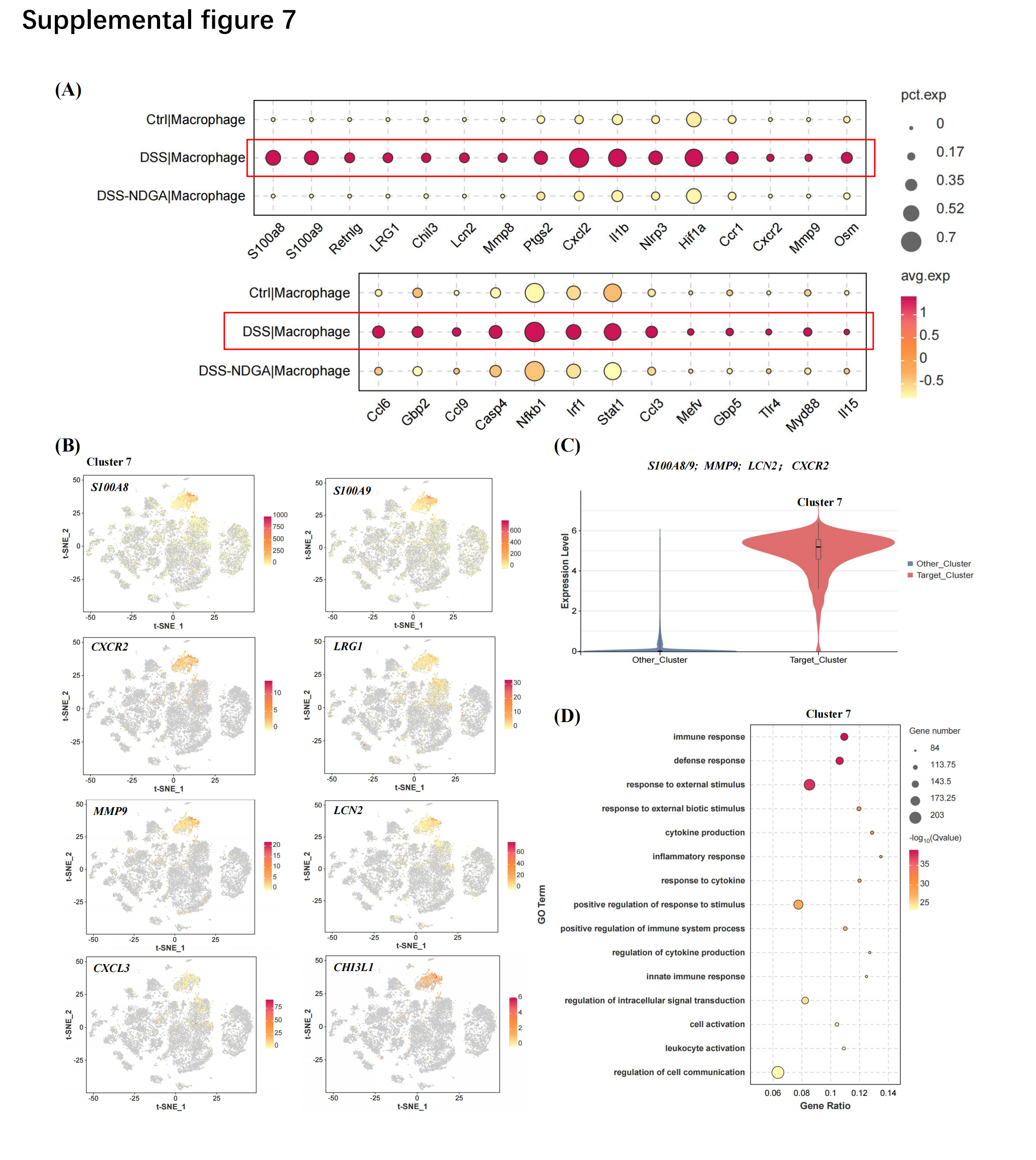
**

**S.Figure 9. NDGA reduced the accumulation of inflammatory genes in cluster 7**

(A) Genes bubble maps of indicated macrophage subsets; (B) tSNE distribution of the Lyz2 and IL-1β-positive cells; (C) violin plot showed the levels of inflammatory genes between cluster 7 and other clusters; (D) GO pathway enrichment analysis of macrophage subsets in cluster 7.

**Supplementary Figure 10**

**
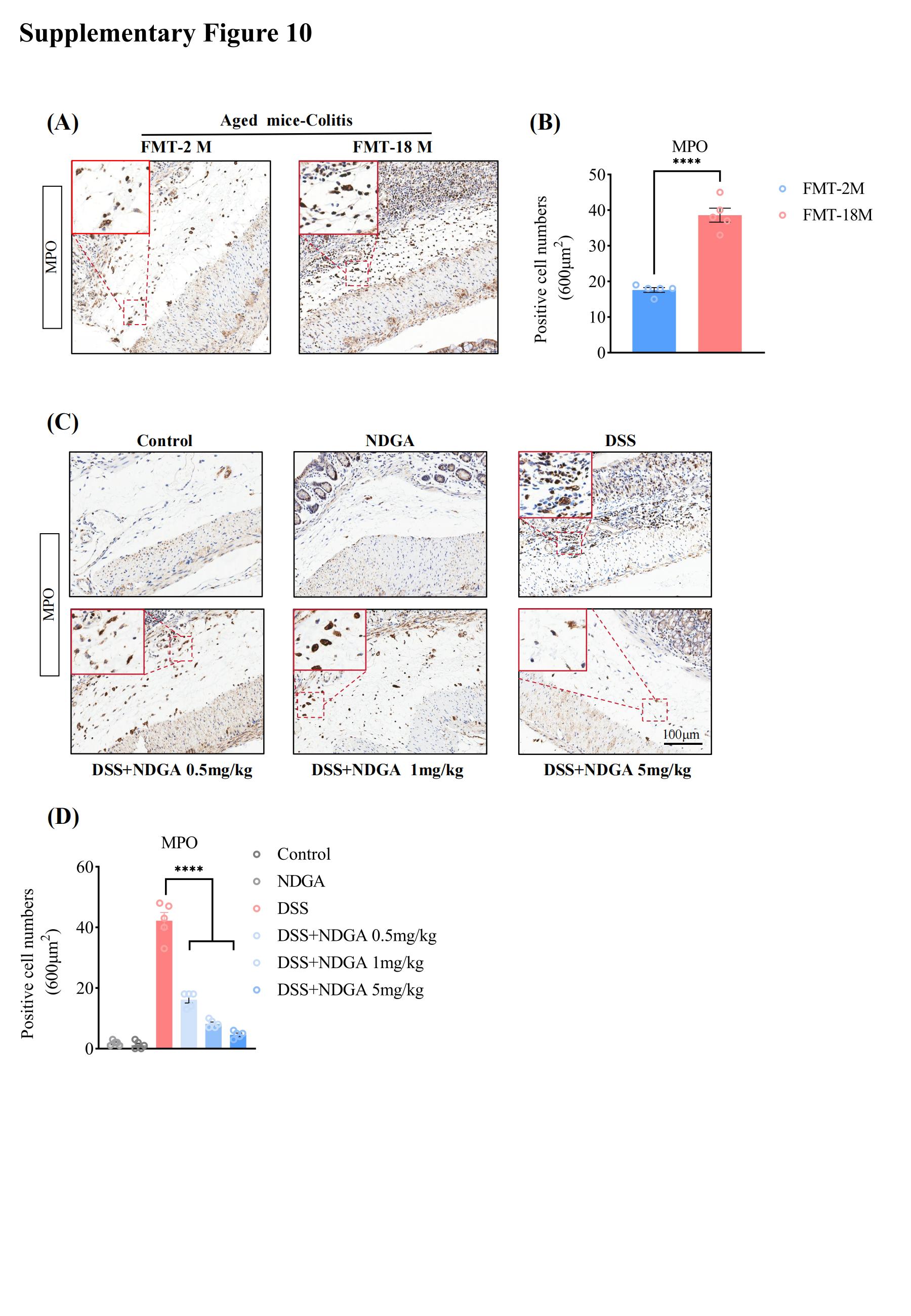
**

**S.Figure 10. Fecal microbiota remodeling and NDGA reduced MPO^+^ neutrophil infiltration in the colon**

The IHC staining of colonic MPO-positive cells and the quantitative analysis of positive cells. (A-B) Aged mice received microbiota from young and aged donors through FMT, followed by treatment with DSS to induce colitis; (C-D) Mice were administered NDGA to prevent DSS-induced colitis.

(Data for each group are expressed as mean ± SEM, n=5. **** p <0.0001; analyzed by one-way ANOVA with Holm-Sidak post hoc tests. Data shown are representative of three independent experiments.)

**Supplementary Figure 11**

**
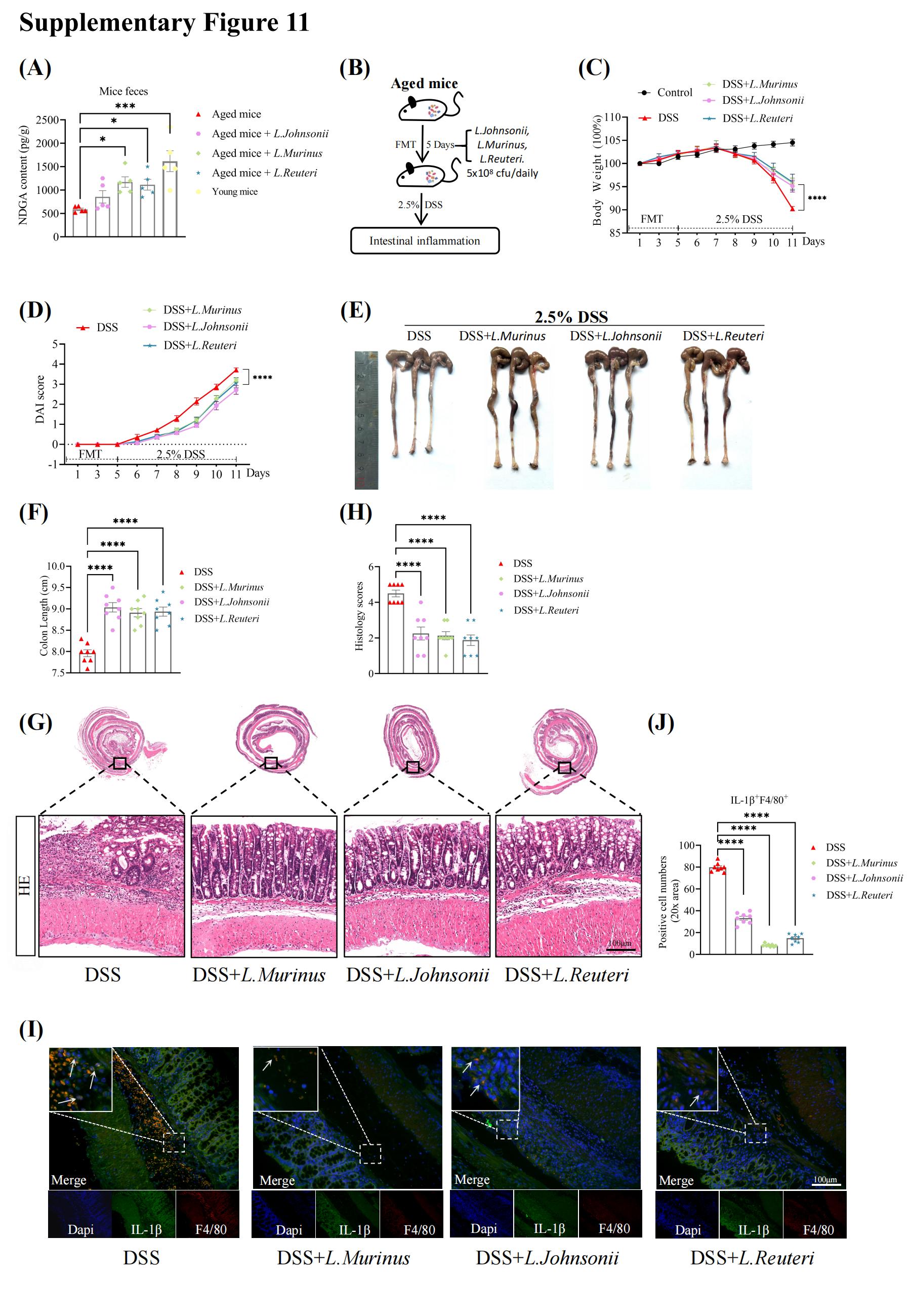
**

**S.Figure 11. Colonization with *Lactobacillus* in aged mice restored fecal NDGA levels and reduced colitis severity**

(A) Fecal NDGA levels were detected by targeted metabolomics in mice with or without *Lactobacillus* colonization; (B) Experimental design of Lactobacillus colonization in aged mice following DSS treatment; (C) Body weight changes were daily monitored after DSS administration; (D) Disease activity index was measured in DSS-treated mice; (E-F) Mice were euthanized on day 7, and colon lengths were measured; (G-H) Representative images of the histological-examined colon sections and the pathological scores were quantified; (I-J) Immunofluorescence detection of F4/80 and IL-1β-positive cells in mouse colon tissues and the quantitative analysis of positive cells;

(Data for each group are expressed as mean ± SEM, n=8. * p <0.05, ** p <0.01, *** p <0.001, **** p <0.0001; analyzed by one-way ANOVA with Holm-Sidak post hoc tests. Data shown are representative of three independent experiments.)

**Supplementary Figure 12**

**
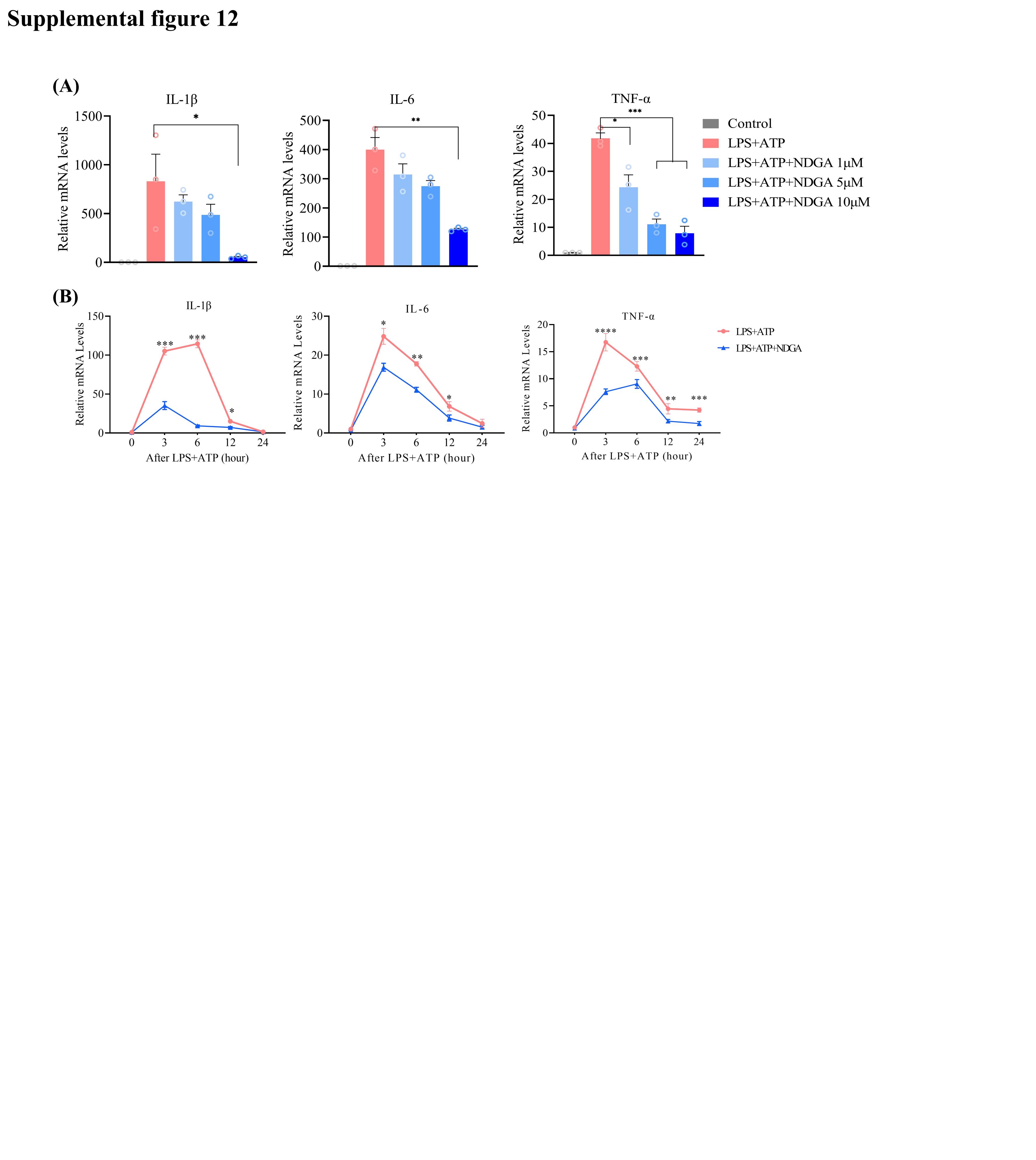
**

**S.Figure 12. NDGA decreases macrophage-released inflammatory mediators upon LPS/ATP stimulation in Raw264.7 cell line**

(A) qRT-PCR detected pro-inflammatory factor levels in the Raw264.7 cell line treated with NDGA at the indicated dosage; (B) qRT-PCR detected pro-inflammatory factor levels in the Raw264.7 cell line treated with NDGA (5μM) at the indicated time.

(Data for each group are expressed as mean ± SEM, n=3. * p <0.05, ** p <0.01, *** p <0.001, **** p <0.0001; analyzed by one-way ANOVA with Holm-Sidak post hoc tests. Data shown are representative of three independent experiments.)

**Supplementary Figure 13**

**
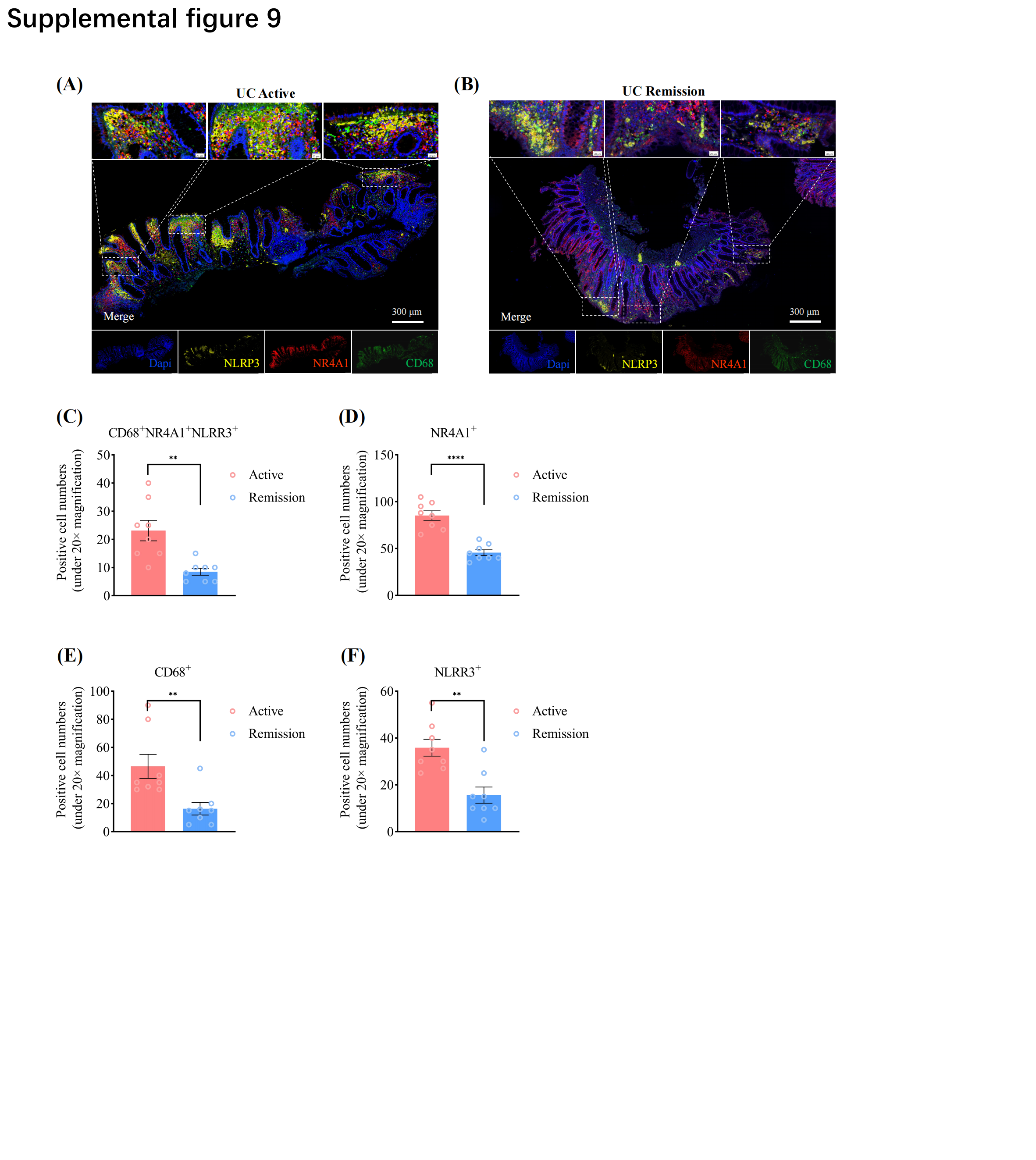
**

**S.Figure 13. Increased infiltration of NR4A1/NLRP3-expressing macrophages in colonic mucosal tissue of active UC patients**

(A-B) Immunofluorescence detection of NR4A1, NLRP3, and CD68-positive cells in the colonic mucosal tissue of patients with UC; (C-F) Counts of CD68-positive, NR4A1-positive, NLRP3-positive, and cells that are triple-positive for NR4A1, NLRP3, and CD68.

(Data in each group are expressed as mean±SEM, UC active and UC remission, n=8. **P<0.01, ****P< 0.0001 by two-tailed Student's *t*-test analysis)

**Supplementary Figure 14**


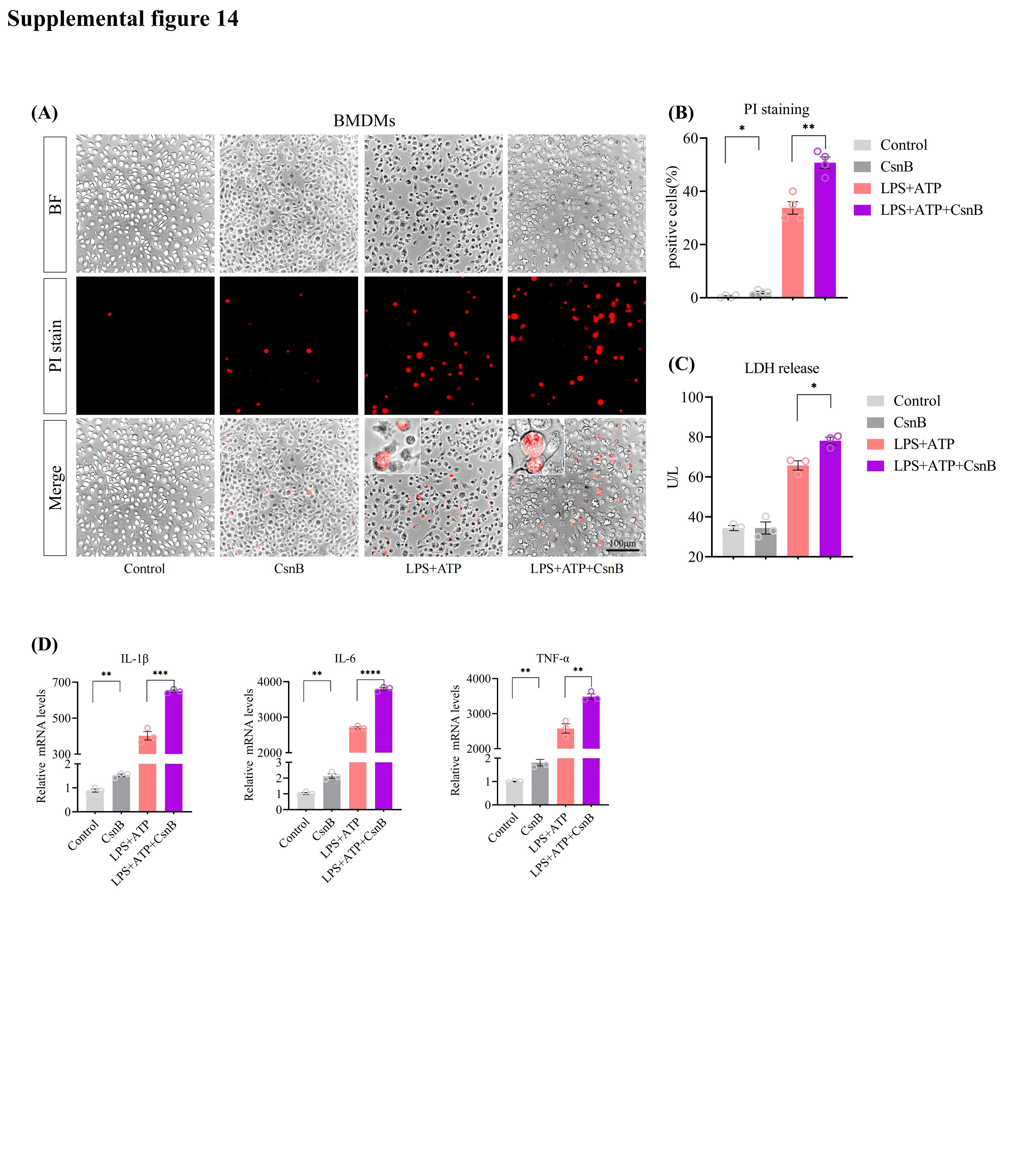


**S.Figure 14. NR4A1 activation promotes LPS/ATP-induced macrophage pyroptosis**

(A-B) PI staining of BMDMs and the quantitative analysis of positive cells; (C) Levels of LDH released from the supernatants of BMDMs; (D) qRT-PCR detected pro-inflammatory factor levels in BMDMs treated with NDGA at the indicated time.

(Data for each group are expressed as mean ± SEM, n=3-4. * p <0.05, ** p <0.01, *** p <0.001, **** p <0.0001; analyzed by one-way ANOVA with Holm-Sidak post hoc tests. Data shown are representative of three independent experiments. CsnB: Cytosporone B, 1μg/ml)

**Supplementary Figure 15**

**
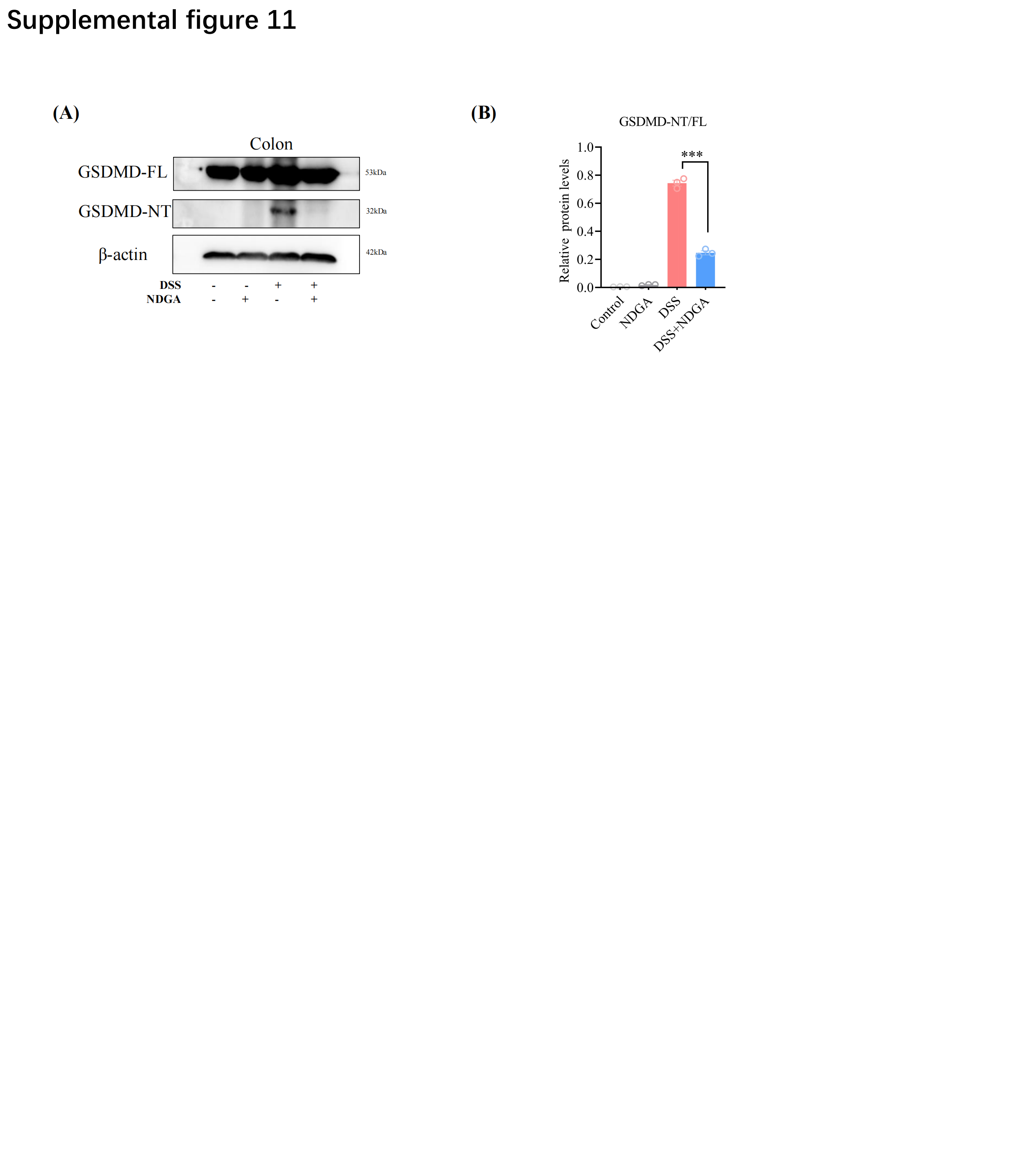
**

**S.Figure 15. NDGA reduces cleaved GSDMD-NT in DSS-treated colon tissue**

(A-B) Immunoblot detection of GSDMD protein levels in colon tissue and their quantitative analysis.

(Data in each group are expressed as mean±SEM, n=3. *** p <0.001; analyzed by one-way ANOVA with Holm-Sidak post hoc tests. Data shown are representative of three independent experiments.)
